# Design and Microwave Absorption Performance Study of SiC-Fe_3_O_4_ Emulsified Asphalt Mixture

**DOI:** 10.3390/ma17163935

**Published:** 2024-08-08

**Authors:** Xiangyu Jiang, Wen Xu, Yixing Chen, Jiaqi Li

**Affiliations:** School of Transportation and Logistics Engineering, Wuhan University of Technology, Wuhan 430063, China

**Keywords:** emulsified asphalt mixture, SiC-Fe_3_O_4_ composite material, mix proportion design, microwave absorption performance, microwave curing

## Abstract

To address the challenges of slow curing speed and suboptimal microwave absorption during the paving of cold-mixed and cold-laid asphalt mixtures, this study introduces SiC-Fe_3_O_4_ composite material (SF) into emulsified asphalt mixtures to enhance microwave absorption and accelerate curing via microwave heating. Initially, based on the maximum density curve theory, an appropriate mineral aggregate gradation was designed, and the optimal ratio of emulsified asphalt mixture was determined through mixing tests, cohesion tests, wet wheel wear tests, and load wheel sand adhesion tests. Subsequently, the influence of SF content on the mixing performance of emulsified asphalt mixtures was analyzed through mixing and consistency tests. Finally, the microwave absorption performance of the mixture was evaluated by designing microwave heating tests under different conditions, using temperature indicators and quality indicators. The experimental results indicate that when SF content ranges from 0% to 4%, the mixing performance of the emulsified asphalt mixture meets specification requirements. The dosage of SF, SF composite ratio, and microwave power significantly impact microwave absorption performance, whereas environmental temperature has a relatively minor effect. The optimal mix ratio for the emulsified asphalt mixture is mineral aggregate:modified emulsified asphalt:water:cement = 100:12.8:6:1. The ideal SF dosage is 4%, with an optimal SiC to Fe_3_O_4_ composite ratio of 1:1, and a suitable microwave power range of 600–1000 W.

## 1. Introduction

Among various grades of highways, asphalt pavement is currently the most extensively used high-grade pavement in China. With the gradual increase in the proportion of road maintenance in the field of road engineering, various road maintenance methods have also been developed. Among these methods, emulsified asphalt mixture is an important material. However, there exists a contradiction between “slow cracking during construction” and “fast setting during molding” in the application of emulsified asphalt [1]. To address this issue, the advantages of microwave heating, which is fast, uniform, and selective, can be utilized to heat emulsified asphalt mixtures [2]. Ordinary emulsified asphalt mixtures have a relatively poor microwave absorption ability [3]. The temperature rise rate after microwave heating is slow, and the water evaporation rate is also relatively slow, which affects the microwave heating curing speed of the mixture.

Based on the relatively poor microwave absorption performance of existing materials in emulsified asphalt mixtures, adding microwave-absorbing materials to improve the microwave absorption performance of the mixture is considered, thereby increasing the microwave curing speed of emulsified asphalt mixtures. Microwave-absorbing materials can be divided into two categories based on their loss mechanisms: electrical loss-type microwave-absorbing materials [4] and magnetic loss-type microwave-absorbing materials [5]. Electrical loss-type microwave-absorbing materials convert the electrical energy of microwaves into thermal energy through electrical loss, while magnetic loss-type microwave-absorbing materials convert the magnetic energy of microwaves into thermal energy through magnetic loss. Both types of materials can be used separately to improve the microwave absorption performance of emulsified asphalt mixtures. Since microwaves are a type of electromagnetic wave that contains both electrical and magnetic energy, the single loss mechanism of single-phase materials cannot fully utilize the electromagnetic energy in microwaves. Therefore, it is necessary to find a microwave-absorbing material that can simultaneously utilize both electrical and magnetic energy.

Wan et al. [6] found through experiments that the main effective components in steel slag that absorb microwaves are iron and Fe_3_O_4_. When Fe_3_O_4_ powder is mixed with mineral powder and steel slag powder of the same quality and size, the temperature rise and heat generated by Fe_3_O_4_ powder are significantly higher than those by iron powder. In samples where both coexist, their contributions to microwave heating are independent, with Fe_3_O_4_ playing a dominant role.

Chen et al. [7] studied the microwave absorption properties of SiC and boron-doped SiC. The results indicated that boron-doped SiC exhibits greater microwave absorption performance in the frequency range of 2–18 GHz compared to SiC. At 14 GHz and a material thickness of 1.5 mm, the maximum microwave reflection loss is −37.94 dB.

Liu et al. [8] proposed that a composite material composed of SiC, an electric loss-type microwave-absorbing material, and Fe_3_O_4_, a magnetic loss-type microwave-absorbing material, can significantly enhance the microwave absorption performance of asphalt mixtures. To improve the efficiency of microwave deicing, researchers designed control specimens of microwave-enhanced functional layer asphalt concrete and asphalt concrete without the microwave-enhanced functional layer. It was found that the microwave-enhanced functional layer significantly improves the microwave heating efficiency and deicing efficiency of asphalt concrete. Through a series of experiments, the optimal ratio of SiC to Fe_3_O_4_ was determined to be 3:1.

Additionally, the research by Liu and Zhao et al. [9,10,11,12] has shown that traditional methods may lead to serious environmental pollution and energy consumption. The microwave heating technology for SF emulsified asphalt mixtures has high microwave energy utilization and is environmentally friendly, and has been widely studied. The research by Li et al. [13] has also demonstrated the excellent microwave absorption capability of Fe_3_O_4_. Microwave heating is currently considered to be the most promising clean energy technology for deicing asphalt pavements.

SiC-Fe_3_O_4_ composite material is a material that combines the characteristics of SiC and Fe_3_O_4_, with potential applications in various fields, including supercapacitors, electromagnetic wave absorption, and thermal management materials. When considering the use of this composite material and its microwave curing method, it is very important to ensure compliance with relevant regulations and safety standards. In articles such as “Microwave heating and deicing efficiency for asphalt concrete with SiC–Fe_3_O_4_ microwave enhanced functional layer” [14] and “Microwave absorption enhancement of asphalt concrete with SiC-Fe_3_O_4_ mixtures modifier” [8], the application of SF in the field of microwave heating of asphalt mixture is mentioned. Research, including the aforementioned articles, shows that the application of SF microwave heating technology is of great significance for extending the service life of road surfaces, reducing maintenance costs, and maintaining environmental protection.

Various microwave-absorbing materials have been proposed, including steel slag, steel wire fiber, carbon fiber, steel wool fiber, iron powder, natural magnetite powder, SiC, Fe_3_O_4_, and SiC-Fe_3_O_4_ binary composite materials. These materials have been studied for their enhancement effects on the microwave absorption performance of asphalt mixtures. Microwave heating technology has been applied in road surface repair, deicing, and curing. A performance comparison of these materials is presented in Table 1.

Based on the current research status both domestically and internationally, this article aims to enhance the microwave absorption performance of emulsified asphalt mixture. A composite material (SF) consisting of the electric loss-type microwave absorption material SiC and the magnetic loss-type microwave absorption material Fe_3_O_4_ is introduced to investigate the microwave absorption performance of emulsified asphalt mixture doped with SF (SF-EAM).

Firstly, the technical performance of various raw materials for emulsified asphalt mixture is evaluated. Secondly, based on the commonly used MS-3 gradation for micro-surface applications, a gradation design is carried out according to the maximum density curve theory. The ratio of the emulsified asphalt mixture is determined through mixing tests, cohesion tests, wet wheel wear tests, and load wheel sand adhesion tests. Subsequently, the influence of SF on the mixing performance of the emulsified asphalt mixture is studied through mixing performance tests, and the reasonable range of SF dosage is determined. Finally, microwave heating experiments are designed with different SF dosages, SF composite ratios, environmental temperatures, and microwave powers. The effects of these factors on the microwave absorption performance of SF-EAM are studied using temperature and quality indicators.

**Figure 1:**
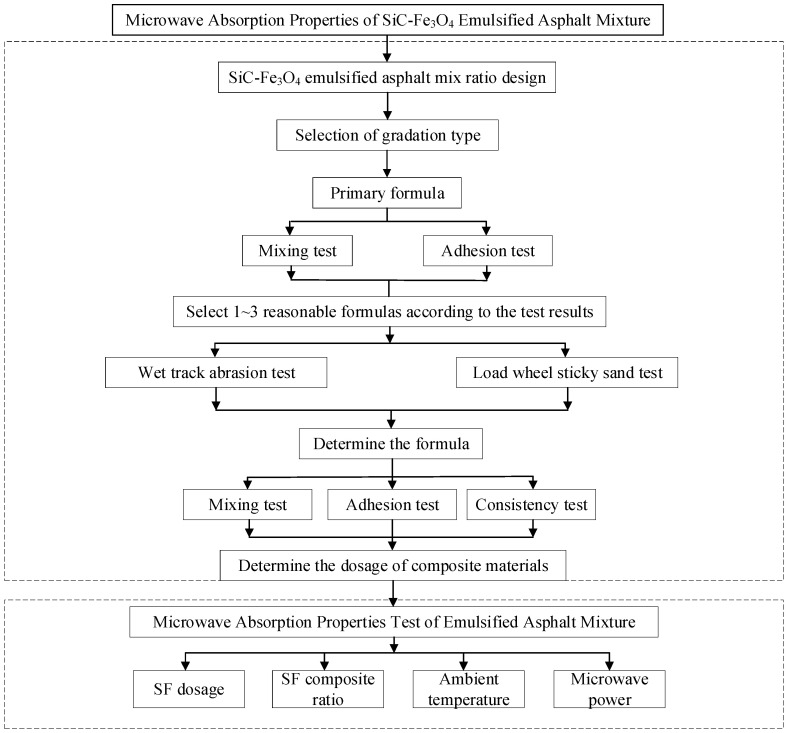
Technical roadmap.

This article employs various methods such as theoretical research and indoor experiments to study the microwave absorption performance of the SiC-Fe_3_O_4_ emulsified asphalt mixture. The technical route is illustrated in Figure 1.

## 2. Materials and Methods

### 2.1. Materials and Preparation

The materials used in this study include modified emulsified asphalt, mineral materials, mineral powder, cement, SiC, and Fe_3_O_4_.

#### 2.1.1. Modified Emulsified Asphalt

The basic performance indicators of the modified emulsified asphalt (self-made) prepared in this study are shown in Table 2.

#### 2.1.2. Mineral Materials

The coarse and fine aggregates (Mazhou Expressway Project Department, Jinzhou, China) used in this study are basalt aggregates. The basic performance indicators of these aggregates are presented in Table 3.

#### 2.1.3. Mineral Powder and Cement

The basic performance indicators of mineral powder (Mazhou Expressway Project Department, Jinzhou, China) used in this study are shown in Table 4. The basic performance indicators of the ordinary Portland cement (Huaxin Cement Co., Ltd., Huangshi, China) used in this study are shown in Table 5.

#### 2.1.4. Preparation of SiC and Fe_3_O_4_ Composite Materials

**Figure 2:**
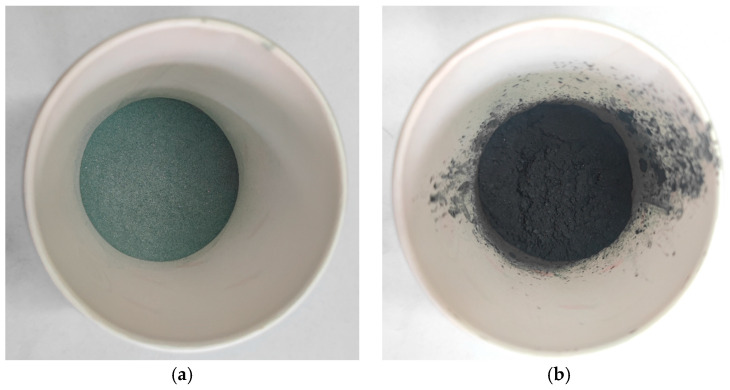
Two types of added materials. (**a**) SiC powder; (**b**) Fe_3_O_4_ powder.

The SiC used in this study is high-purity silicon carbide powder produced by Nangong Yingtai Metal Materials Co., Ltd. (Xingtai, China). The SiC content in the silicon carbide powder is greater than 99%, and the appearance of the silicon carbide powder is shown in Figure 2a. The basic technical performance of SiC is evaluated according to the technical requirements for mineral powder, with the test results presented in Table 6.

The Fe_3_O_4_ used in this study is high-purity iron oxide powder produced by Hebei Keze Metal Materials Co., Ltd. (Xingtai, China). The Fe_3_O_4_ content in the iron oxide powder is greater than 99%. The appearance of the iron oxide powder is shown in Figure 2b. The basic technical performance of Fe_3_O_4_ is evaluated according to the technical requirements for mineral materials, with the test results presented in Table 7.

**Figure 3:**
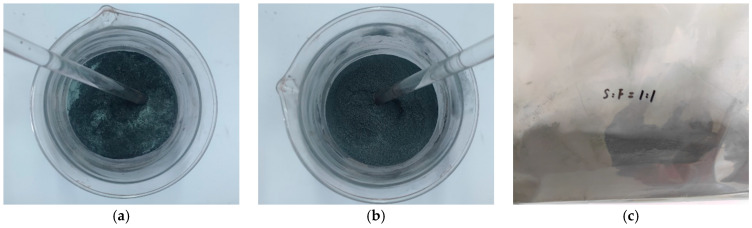
Preparation of SiC-Fe_3_O_4_ composite material. (**a**) Mix (**b**) thoroughly and evenly; (**c**) bag for temporary storage.

Based on the characteristics of the two materials having similar densities and no chemical reactions with each other [25,26], the method for preparing SiC-Fe_3_O_4_ composite materials from SiC and Fe_3_O_4_ in this study is as follows: a specified mass of SiC powder and Fe_3_O_4_ powder are mixed in a stirring cup, stirred thoroughly with a glass rod, and once the two materials are completely and evenly mixed, they are placed in a sealed bag for later use, as shown in Figure 3.

### 2.2. Test Methods

#### 2.2.1. Mixing Test

The oil-to-stone ratio range specified in China’s standards for MS-3 micro-surfacing is 6.0% to 8.5% [22]. Calculated based on a 60% evaporation residue content, the corresponding emulsified asphalt range is 10% to 14.2%. Based on this range, the initial selection of emulsified asphalt dosage in the mixing test is 13%, corresponding to an oil-to-stone ratio of 7.8%. Cement, used as a filler, has a small dosage and a relatively minor impact on the mix design of the emulsified asphalt mixture; thus, a commonly used dosage of 1% is adopted. The particle sizes of the added SiC and Fe_3_O_4_ materials are both less than 0.075 mm. In the mix design process, the role of SF is similar to that of mineral powder, and their influence on the initial mix ratio is not considered. The amount of water added is determined according to the consistency of the mixture. Since the asphalt emulsion contains some water, based on existing experiments, when the amount of modified emulsified asphalt is 13%, the range of added water should be 3% to 7%.

**Figure 4:**
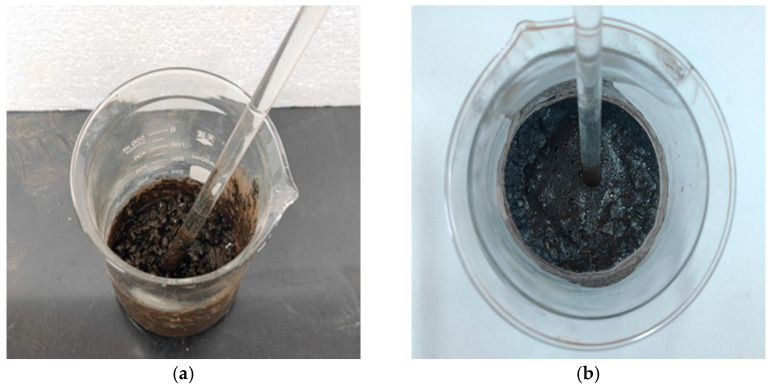
Mixing test. (**a**) The state when the mixture begins to thicken; (**b**) when there is too much water added.

The mixing test procedure is shown in Figure 4, with the specific steps as follows:

1. Prepare a certain quantity of mineral materials, modified emulsified asphalt, cement, water, and other materials. Divide the test materials into 5 groups, pour the materials into the mixing cup one by one, and start timing.
2. Mix the mixture evenly. During the mixing test, when the mixture begins to thicken and feels strong in the hands, record the time at this moment. This time is the mixable time of the mixture. Record the mixing time for the 5 groups of emulsified asphalt mixtures in sequence, accurate to 1 s.

#### 2.2.2. Cohesion Test

**Figure 5:**
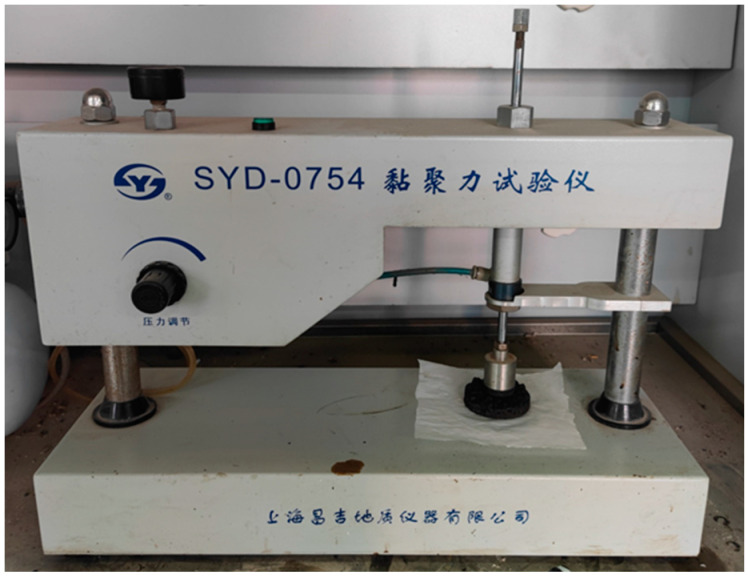
Cohesion Tester.

The cohesion tester (Changji Geological Instrument Co., Ltd., Shanghai, China) used in this study is shown in Figure 5. After determining the appropriate amount of added water for the emulsified asphalt mixture through mixing experiments, this amount was selected for the cohesion tests. A series of cohesion tests were conducted by fixing the quantities of other materials and only varying the amount of emulsified asphalt. The optimal amount of emulsified asphalt was selected based on the measured cohesion values and the failure state of the test specimens.

According to the Technical Guidelines for Micro-Surfacing and Slurry Seal [22] (hereinafter referred to as the “Guidelines”), the actual oil-to-stone ratio used for MS-3 grading is typically between 6.0% and 7.5% and should not exceed the specified limit of 8.5%. Therefore, based on a 60% evaporation residue content, the dosage range of modified emulsified asphalt was selected to be 10% to 14%, corresponding to an oil-to-stone ratio range of 6% to 8.4%. The dosage ratios of various materials used in the cohesion test are summarized in Table 8.

The specific steps for the cohesion test are as follows:

1. Prepare a certain quantity of mineral materials, modified emulsified asphalt, cement, water, and other materials. Group the test materials.
2. Mix the materials and prepare the test specimens, then demold and start timing. Measure the cohesion of the specimens after 30 min and 60 min of curing for the 5 groups, respectively, and record the failure state of the specimens.

#### 2.2.3. Wet Wheel Wear Test

**Figure 6:**
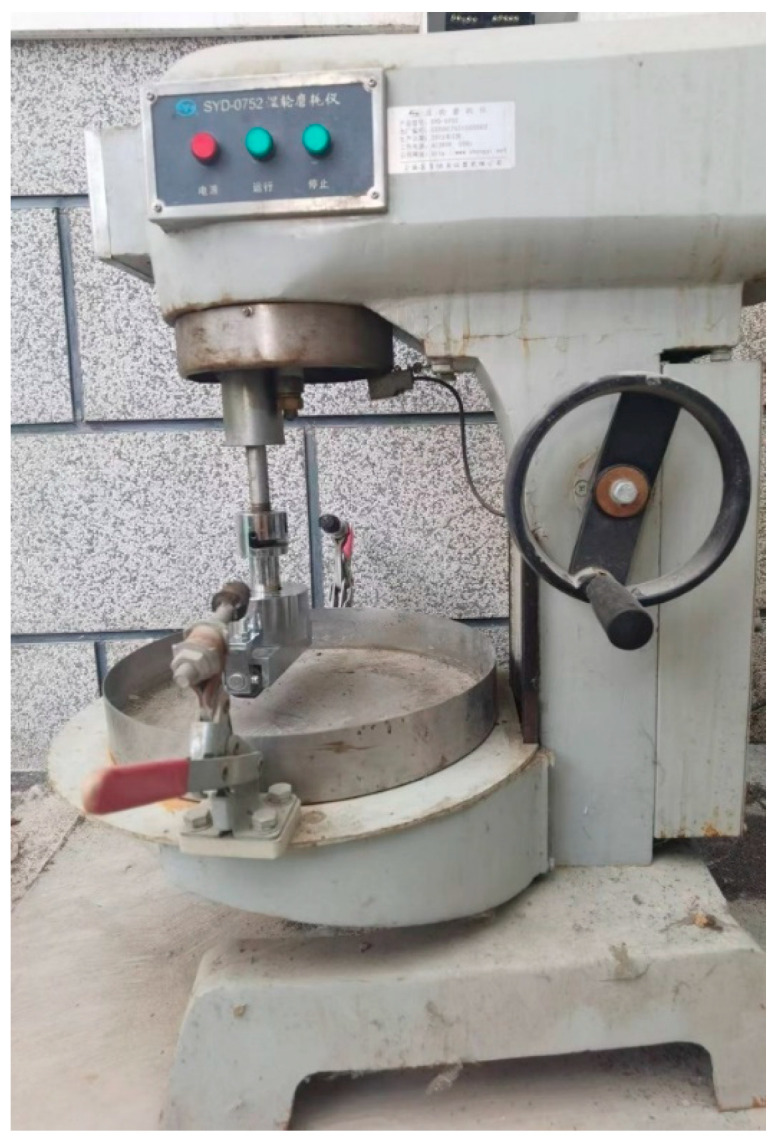
Wet Wheel Abrasion Tester.

The wet wheel abrasion tester (Changji Geological Instrument Co., Ltd., Shanghai, China) used in this study is shown in Figure 6. The steps for conducting the wet wheel wear test are as follows:

1. Weigh the total mass of the oil felt and the test piece.
2. Place the prepared test piece and oil felt in a water bath at 25 °C for 1 h.
3. Install the sample in the tester and start the instrument.
4. After the test is completed, dry the oil felt and test piece to a constant weight and then weigh the total mass.

The calculation method for the wear value is shown in Equation (1).

(1) $$WTAT=(m_{a}-m_{b})/A$$

where

WTAT is the wear value of slurry mixture, g/m^2^;

$m_{a}$ is the weight of the specimen before wear, g;

$m_{b}$ is the weight of the worn specimen, g;

*A* is the wear surface area of rubber hose with wear head, m^2^.

#### 2.2.4. Load Wheel Sand Adhesion Test

**Figure 7:**
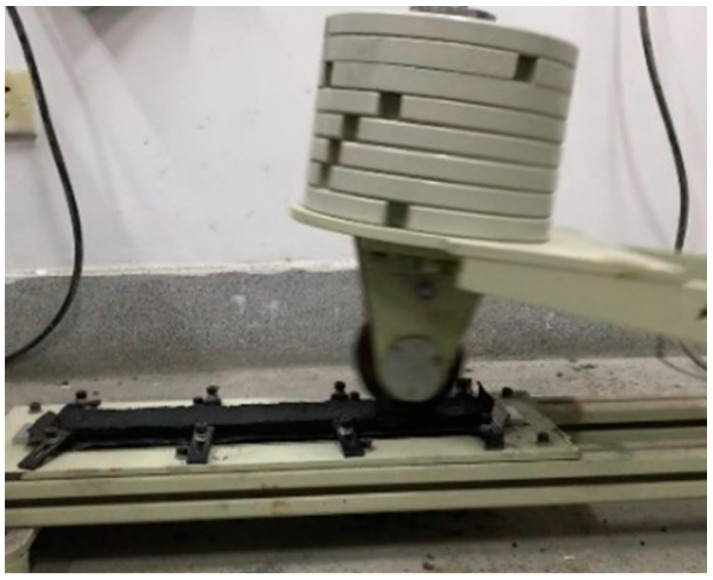
Load wheel sand adhesion tester.

The load wheel sand adhesion tester (Changji Geological Instrument Co., Ltd., Shanghai, China) used in this study is shown in Figure 7. The steps for conducting the load wheel sand adhesion test are as follows:

1. Install the sample in the tester and start the instrument.
2. Roll the sample 1000 times at a frequency of 44 times per minute.
3. Dry and cool the sample, then weigh the mass (*G_a_*).
4. Reinstall the sample, lay the preheated sand flat on the sample, and place the cover plate.
5. Roll the machine an additional 100 times, brush off the floating sand, and weigh the mass (*G_b_*).

The calculation method for the adhesive sand quantity is shown in Equation (2).

(2) $$LWT=(G_{b}-G_{a})/A$$

where

*LWT* is the adhesive sand content of the emulsified asphalt, g/m^2^;

*A* is the rolling area, m^2^;

*G_a_* is the mass of the specimen after 1000 rounds of rolling, g;

*G_b_* is the quality of the specimen after 100 rounds of sanding and rolling, g.

#### 2.2.5. Consistency Test

**Figure 8:**
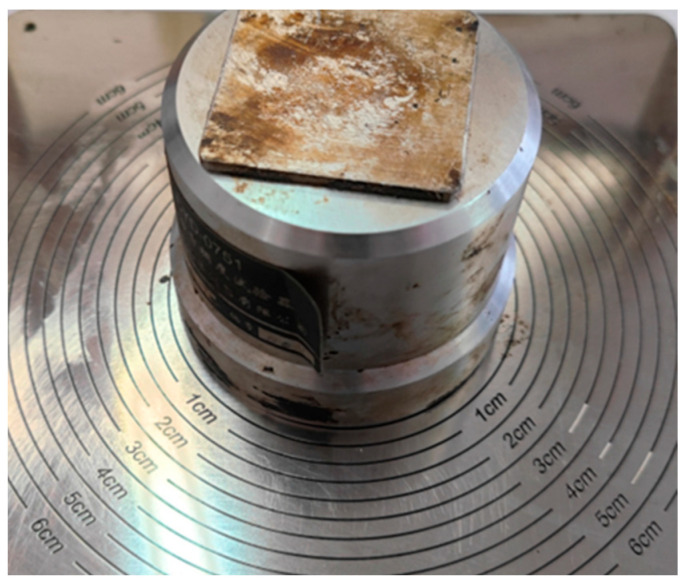
Consistency tester.

The consistency tester (Changji Geological Instrument Co., Ltd., Shanghai, China) used in this study is shown in Figure 8. The consistency test determines the consistency of the emulsified asphalt mixture, which can be affected by the addition of SF, altering the slurry state of the mixture. Both ASTM and ISSA specify that the viscosity of slurry mixtures should be within the range of 2–3 cm [22]. The viscosity of the emulsified asphalt mixtures with different mass fractions of SF is determined through viscosity tests, and the reasonable range of SF dosage is identified based on the viscosity specifications.

The specific steps for the consistency test are as follows:

1. The material preparation process is the same as in the mixing test. Pour the raw materials into the mixing pot in sequence and mix well. Then, pour the emulsified asphalt mixture into the cone of the consistency meter and cover it with a bottom plate with a scale.
2. Invert the cone and base plate together, then immediately lift the cone upwards to allow the mixture to collapse naturally.
3. Measure the distance between the edge and the central circular edge of the collapsed emulsified asphalt mixture, which is the consistency of the mixture. Measure the consistency of 6 sets of emulsified asphalt mixtures in sequence, accurate to 0.1 cm.

#### 2.2.6. SF-EAM Microwave Absorption Performance Test

**Figure 9:**
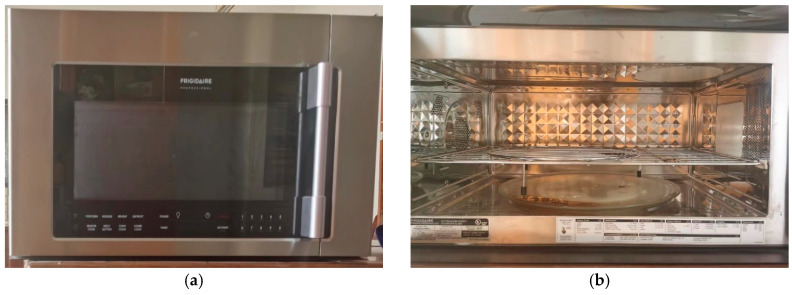
Microwave oven. (**a**) Appearance; (**b**) internal structure.

The appearance and interior of the microwave oven (Frigidaire, Zhongshan, China) used in this study are shown in Figure 9. The microwave oven’s dimensions are 0.74 m in length, 0.40 m in width, and 0.44 m in height. The internal cavity size is 0.53 m in length, 0.36 m in width, and 0.25 m in height. The waveguide is located on the right side of the microwave oven, suitable for heating common emulsified asphalt mixture specimens. The maximum heating power of the microwave is 1000 W, with a frequency of 2.45 GHz, and the minimum accuracy of microwave heating time is 1 s. The standard curing box has a curing temperature range of 0–85 °C and a curing relative humidity range of 45–98%. The maximum range of the electronic balance is 1000 g, with a minimum accuracy of 0.001 g. Other instruments and equipment include mixing pots, mixing rods, glass cups, measuring cylinders, gloves, and wooden boards.

The aggregate proportion of coarse and fine aggregates is approximately 89%, and the proportion of mineral powder is approximately 11%. Therefore, a total of 2225 g of coarse and fine aggregates, 225 g of mineral powder, 320 g of modified emulsified asphalt with an oil-to-stone ratio of 7.7%, 150 g of water, 25 g of cement, and 25 g of SiC powder and Fe_3_O_4_ powder with a particle size of 0.07 mm were weighed. These materials were divided into 5 groups. In each group, a certain mass of SiC powder and Fe_3_O_4_ powder were first combined to form SF, which then replaced some of the mineral powder.

**Figure 10:**
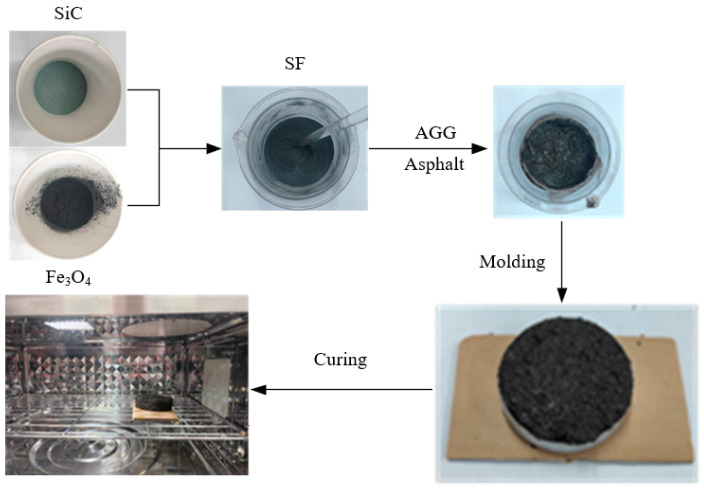
SF-EAM specimen preparation and heating process.

All specimens were prepared in a laboratory at a specific ambient temperature and relative humidity of 75%, as follows:

1. First, mix the minerals, cement, and SF in each mixture and stir evenly. Then, add water to the mixture and mix well. Finally, add modified emulsified asphalt and stir for 30 s.
2. Divide the mixed emulsified asphalt mixture into two equal parts and pour them into two identical circular test molds to make two types of identical specimens. The inner diameter of the test mold is 80 mm, and the height is 20 mm. The two types of specimens are used for temperature testing and quality testing, respectively.
3. Compact and scrape the mixture to ensure a mass of 225 g for each specimen, then demold the specimen and place it in a 25 °C curing box for 10 min. Prepare 5 sets of specimens in sequence according to the above method, as shown in Figure 10.

**Figure 11:**
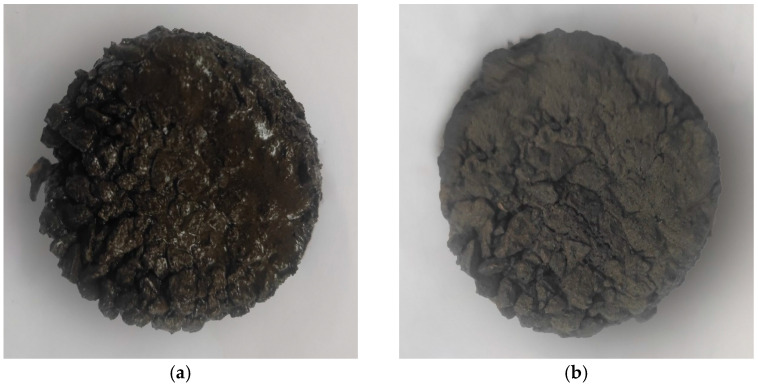
Comparison of specimens before and after microwave heating. (**a**) Before heating; (**b**) after heating.

**Figure 12:**
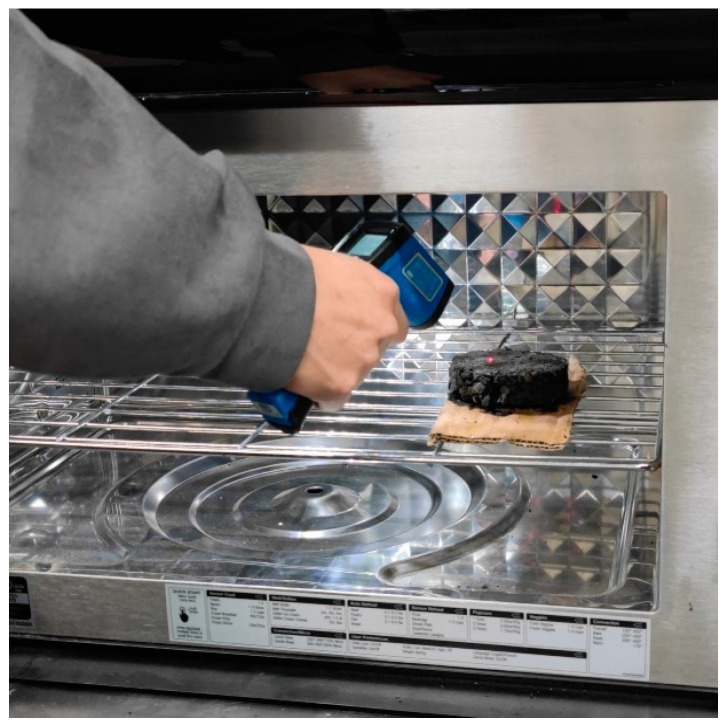
Temperature test.

In the temperature-testing experiment, a specific microwave power and an initial temperature of the specimen are used. Three measuring points on the surface of the specimen are selected, and their surface temperatures are measured using an infrared thermometer (TASI, Suzhou, China). The average temperature of the three measuring points is calculated and recorded as the surface average temperature. The methodology is as follows: Place the specimens used for temperature testing in Group 1 in the microwave oven, turn on the microwave oven to heat for 140 s, and pause to measure the surface temperature of the specimens every 20 s, accurate to 0.1 °C. Heat and test 5 sets of specimens in sequence. The specimens before and after microwave heating are shown in Figure 11. The testing process of the temperature test is shown in Figure 12.

**Figure 13:**
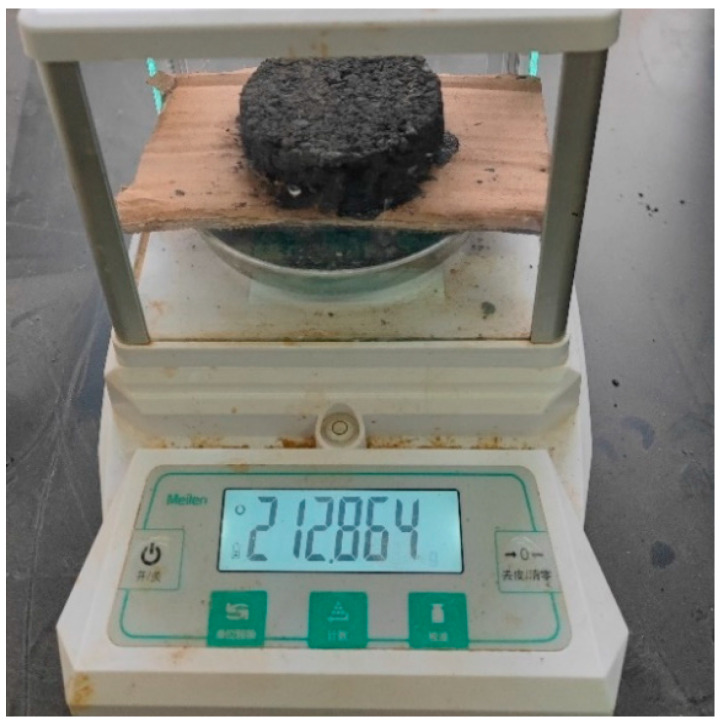
Quality Testing.

For the quality testing experiment, a specific microwave power and initial temperature of the specimen are used. The methodology is as follows: Place the specimens used for quality testing in Group 1 in the microwave oven, turn on the microwave oven, and heat for 140 s. Measure the mass of the specimens every 20 s using an electronic balance, accurate to 0.1 g. Heat and test 5 sets of specimens in sequence, and the testing process for quality testing is shown in Figure 13.

## 3. Results and Discussion

### 3.1. Mix Proportion Design of SiC-Fe_3_O_4_ Emulsified Asphalt Mixture

In road maintenance engineering, emulsified asphalt mixture is often used in micro-surfacing technology, and the mix design of emulsified asphalt mixture is based on the “Guidelines” [22]. First, technical performance tests on raw materials are conducted. The type and range of aggregate gradation are determined based on road usage requirements, and the gradation curve is adjusted according to gradation design theory to design a suitable gradation. Next, based on existing experience in designing mix proportions, the amount of water added is determined through mixing tests, and the range of oil-to-stone ratio is determined through cohesion tests. One to three reasonable mix proportions are initially selected. Then, the optimal oil-to-stone ratio is determined through wet wheel wear tests and load wheel sand adhesion tests, and the final mix ratio of the mixture is determined. Finally, SF is used to replace some mineral powder, and the reasonable dosage range of SF is determined based on the mixing performance requirements of emulsified asphalt mixtures specified in the “Guidelines” [22] and through mixing tests and consistency tests.

#### 3.1.1. Mix Proportion Design of Emulsified Asphalt Mixture

The emulsified asphalt mixture prepared in this study is mainly used in the maintenance engineering of high-grade pavements. The grading type should be MS-3 continuous grading. Additionally, considering that high-grade highway pavements need to have excellent anti-rutting performance, coarse aggregates should be selected for design.

According to Fuller’s theory [27], when studying the relationship between the degree of overlap between grading curves and parabolic shapes and the compactness of their mixtures, it was found that the higher the degree of overlap, the greater the compactness of the mixture. The resulting Fuller formula is shown in Equation (3). Based on practical construction conditions, Taibo improved the Fuller formula and derived the Taibo formula, as shown in Equation (4). According to research by relevant scholars [27], when the value is small, the flowability of the emulsified asphalt mixture is good; in this study, it is set at 0.4.

(3) $$P=100\times \sqrt{\frac{d}{D}}$$

(4) $$P=100\times {\left(\frac{d}{D}\right)}^{n}$$

where

*P* is the percentage of aggregate passing, %;

*d* is the sieve size, mm;

*D* is the maximum particle size of the aggregate, mm;

*n* is the experimental index.

In summary, based on the MS-3 grading, this section calculates the grading pass rate using the Taibo formula with *n* = 0.4, and compares it with the median value of the MS-3 grading. The cumulative pass rate data of the two grading groups are shown in Table 9. According to the cumulative pass rate data of particle size distribution in Table 9, the pass rate between the sieve hole particle sizes of the two groups of gradations is calculated. The difference and variation amplitude of the pass rate between the two groups are compared, as shown in Table 10.

Based on the difference and ratio data calculated in Table 10, the following can be observed:

1. When the particle size range of the mixture is 4.75~9.5 mm, the pass rate of the median in the MS-3 grading is lower than that obtained by the Taibo formula at *n* = 0.4, indicating that the proportion of large-sized coarse aggregates in the median of the MS-3 grading is relatively small.
2. When the particle size range of the mixture is 0.6~4.75 mm, the pass rate of the median in the MS-3 grading is greater than that obtained by the Taibo formula at *n* = 0.4 with accounting for a large proportion. Specifically, in the range of 1.18~2.36 mm, the difference accounts for 33%, indicating that the proportion of medium and small-sized coarse aggregates and large-sized fine aggregates in the median of the MS-3 grading is relatively high.
3. When the particle size range of the mixture is 0.075–0.6 mm, the pass rate of the median in the MS-3 grading is lower than that obtained by the Taibo formula at *n* = 0.4. Although the absolute difference is not significant, it accounts for a large proportion, especially in the range of 0.075–0.15 mm, where the difference accounts for as much as 46%, indicating that the proportion of small particle-sized fine aggregates in the median of the MS-3 grading is relatively small.

**Figure 14:**
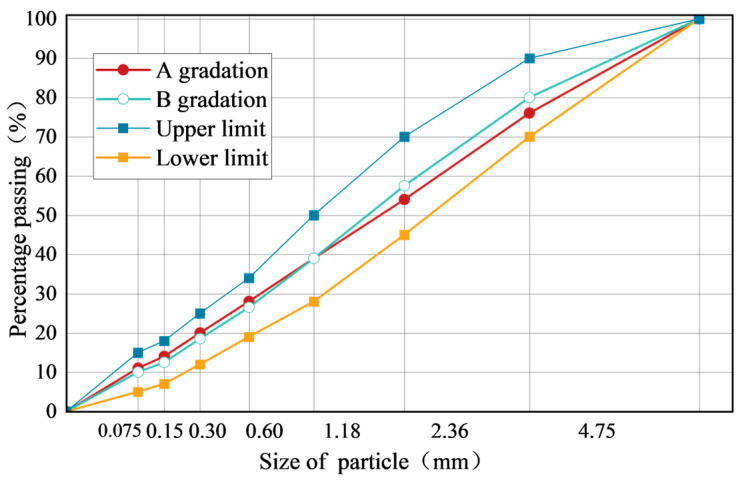
Grading curve.

Based on the above requirements, the final grading A for this study was designed. The grading curve is depicted in Figure 14.

After initially selecting two types of mineral aggregate gradations, and combining them with existing experience in emulsified asphalt mixture ratios for micro-surfacing, the dosage of emulsified asphalt, cement, and water is preliminarily selected. The range of commonly used materials for emulsified asphalt mixtures as specified in the “Guidelines” [22] is shown in Table 11. The mixing time and cohesion specifications for the mixing test and cohesion test are provided in Table 12.

Within the specified range of material usage, emulsified asphalt mixture specimens were prepared based on empirical mix proportions for mixing and cohesion tests. The amount of added water was determined through mixing tests, and the range of the oil-to-stone ratio was determined through cohesion tests. Based on the results of these tests, gradation was selected, and 1–3 reasonable mix proportions were preliminarily chosen.

**Figure 15:**
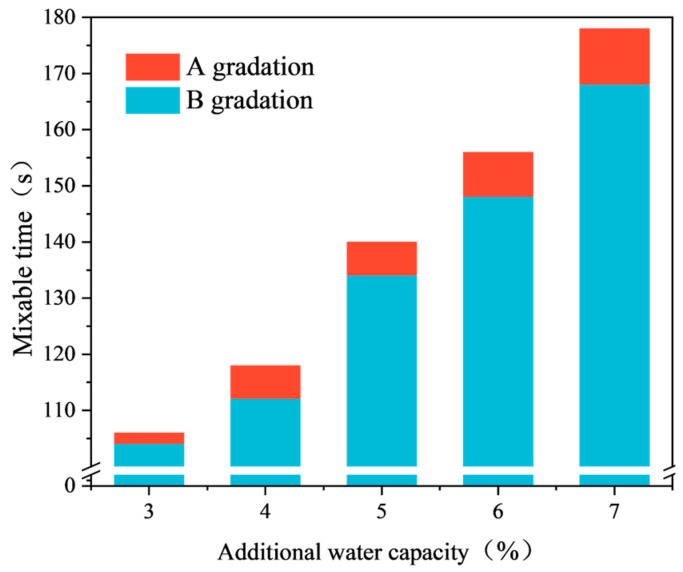
Mixing time corresponding to different added water amounts.

The test results of the mixing test under two different gradations are shown in Figure 15.

Based on the experimental data in Figure 15, the following observations were made:

- When the external water content is less than 4%, the mixing time of the emulsified asphalt mixture under both gradations is less than 120 s, which does not meet the specification requirements.
- When the external water content is greater than 5%, the mixing time exceeds 120 s, meeting the specification requirements.
- When the external water content reaches 7%, the surface of the emulsified asphalt mixture becomes relatively moist, with some flowing water, making it difficult to form a cohesive mixture.
- Under the same moisture content conditions, the mixing time for gradation A is higher than for gradation B, with an average increase of about 5%.

When the amount of external water is small, the fluidity of the emulsified asphalt mixture is insufficient, and the aggregate cannot be fully mixed with the emulsified asphalt. Conversely, when the amount of external water is too high, although the mixing performance improves, the emulsified asphalt mixture specimen system becomes loose and cannot be quickly formed during specimen preparation.

In Gradation B, the content of coarse aggregate is relatively low, while the proportion of fine aggregate is high. Due to the large specific surface area of the fine aggregate, more water from the asphalt emulsion is absorbed onto the aggregate surface during the wetting preparation stage. This process reduces the amount of free water in the mixing system, leading to a decrease in the fluidity of the mixture. Consequently, the mixing time for Gradation B is relatively short.

Considering the experimental results of the mixing tests under the two different gradations, it is proposed that the external water content for the emulsified asphalt mixture be set at 6%.

**Figure 16:**
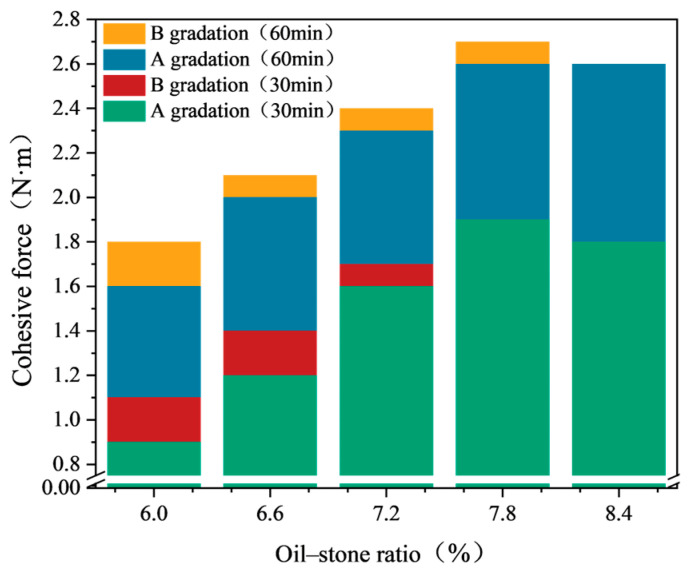
Cohesion force corresponding to different oil–stone ratios.

The test results of the cohesion test under two different gradations are shown in Figure 16. According to the experimental data in Figure 16, when the oil-to-stone ratio is 6%, the 30-min cohesion of the emulsified asphalt mixture under both gradations is less than 1.2 N·m, and the 60-min cohesion is less than 2.0 N·m, which does not meet the specification requirements. As the oil-to-stone ratio increases, the cohesion of both groups of mixtures also increases. When the oil-to-stone ratio exceeds 6.6%, the cohesion meets the specification requirements. However, when the oil-to-stone ratio reaches 8.4%, the cohesion of the mixture shows a decreasing trend.

When the oil-to-stone ratio is small, the cohesion of the emulsified asphalt mixture with Gradation B is slightly higher than that with Gradation A. But when the oil-to-stone ratio exceeds 7.8%, the cohesion of the two groups of mixtures becomes close. When the oil-to-stone ratio is relatively small, there is insufficient emulsified asphalt in the mixture to bond with the aggregate, resulting in lower cohesion due to weak bonding between aggregate particles. As the emulsified asphalt content increases, the bonding between the emulsified asphalt and aggregate improves, leading to increased cohesion. When the emulsified asphalt content is too high, the amount of unbound asphalt in the system increases, causing the cohesion to decrease. Gradation B contains relatively fine aggregate with fewer voids in the mixture, resulting in a stronger overall bonding effect and higher cohesion.

Taking into account the test results of cohesion tests under the two different gradations, the proposed range for the oil-to-stone ratio in emulsified asphalt mixtures is 6.6% to 8.4%. Based on the results of the mixing test and cohesion test, it can be concluded that, under the premise that both sets of gradation test technical indicators meet the specification requirements, the gradation of the emulsified asphalt mixture applied to the micro-surfacingshould be selected as coarse type. Therefore, Gradation A is chosen as the final gradation for this study. The Preliminary proportions of emulsified asphalt mixtures and design indicators for wet wheel wear test and load wheel sand adhesion test as specified in the “Guidelines” [22] is shown in Table 13 and Table 14.

**Figure 17:**
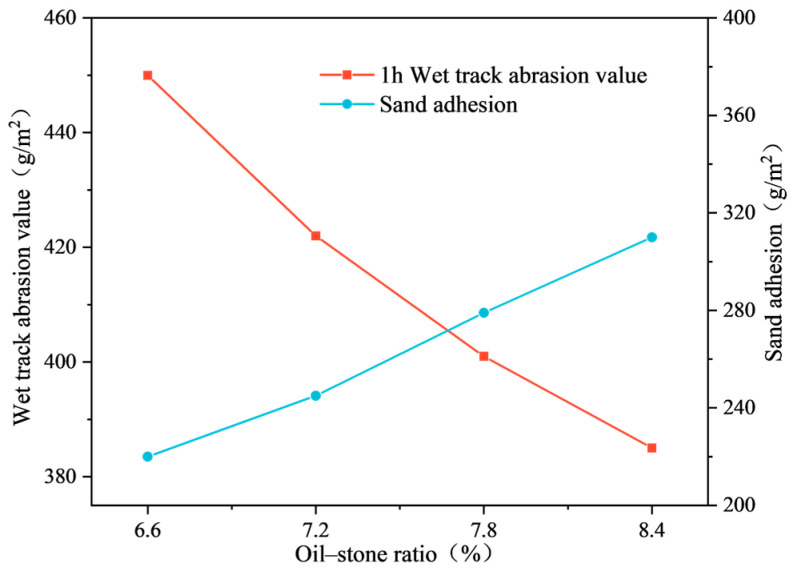
Relationship curve between wear value, adhesive sand content, and oil–stone ratio.

The test results of the wet wheel wear test and load wheel sand adhesion test are shown in Figure 17. As illustrated in Figure 17, with the increase in the oil-to-stone ratio, the content of emulsified asphalt continues to rise. Consequently, the wet wheel wear curve decreases continuously, while the adhesion sand curve increases continuously. The two curves intersect in the graph, and the oil-to-stone ratio corresponding to this intersection point is the optimal oil-to-stone ratio.

The intersection point in the figure indicates an oil-to-stone ratio of approximately 7.7%. The corresponding 1-h wet wheel wear value is 405 g/m^2^, which is lower than the specification requirement of 540 g/m^2^. The corresponding load wheel adhesion sand amount is 275 g/m^2^, which is less than the specification requirement of 450 g/m^2^. Both indicators meet the specification requirements. Therefore, the final oil-to-stone ratio of the emulsified asphalt mixture was determined to be 7.7%, corresponding to a modified emulsified asphalt dosage of 12.8%.

In summary, the final ratio of the emulsified asphalt mixture is determined as follows: mineral aggregate:modified emulsified asphalt:water:cement = 100:12.8:6:1, with Grade A used for the mineral aggregate.

#### 3.1.2. Content Range of SiC-Fe_3_O_4_ Composite Materials

By conducting mixing and consistency tests, the mixing performance of the emulsified asphalt mixture mixed with SF (SiC-Fe_3_O_4_ composite materials) was studied. The reasonable dosage range of composite materials was determined based on the test results. The mixing time and consistency requirements specified in the guidelines are shown in Table 15.

The optimal ratio of emulsified asphalt mixture determined earlier is mineral aggregate:modified emulsified asphalt:water:cement = 100:12.8:6:1. The particle size of the SiC and Fe_3_O_4_ materials used to replace the mineral powder is mostly concentrated in the range of 0.06~0.075 mm. The MS-3 graded emulsified asphalt mixture specifies a range of 5%~15% for mineral materials with a particle size below 0.075 mm [22]. Therefore, in the mixing performance test of the emulsified asphalt mixture, the initial range of SF dosage is set at 0~5%. Due to the small difference in particle size between the two materials, the influence of the SF composite ratio on mixing performance can be ignored. Therefore, in the mixing performance test, the SF composite ratio is set at 1:1. Additionally, environmental conditions significantly impact the mixing performance of emulsified asphalt mixtures. To minimize the influence of environmental factors, the mixing performance tests of the mixture were completed in a windless laboratory environment with a temperature of 25 °C and relative humidity of 75%.

**Figure 18:**
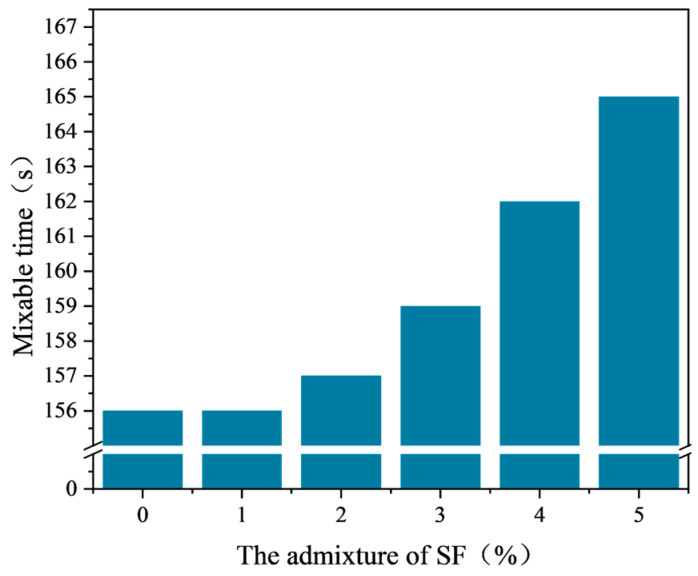
Mixing time of emulsified asphalt mixture with different SF content.

After the mixing test, the mixing time of emulsified asphalt mixtures with different SF contents is shown in Figure 18. According to the experimental data in Figure 18, when the SF content is within the range of 0% to 5%, the mixing time of the emulsified asphalt mixture is greater than 120 s, meeting the specification requirements. When the SF content is 1%, the mixing time is the same as that of the mixture without SF. When the SF content is within the range of 1% to 5%, the mixing time increases continuously with the increase in SF content. When the SF content reaches 5%, the mixing time is 165 s, an increase of 9 s compared to the absence of SF.

The particle size range of SF is mostly distributed around 0.06~0.075 mm, while the particle size of the replaced mineral powder is widely distributed between 0~0.075 mm. At low dosages, a small amount of SF will not affect the gradation of the mixture significantly, so its impact on the mixing time is also relatively small. As the SF content increases, the content of coarse particles in the emulsified asphalt mixture also increases. Coarse SF particles have a smaller specific surface area than fine particles in the mineral aggregate, reducing the ability of particles to adsorb water. As a result, the amount of free water increases, leading to a longer mixing time. Additionally, SF particles are mostly uniformly distributed with fewer pores than mineral powder particles, resulting in lower water absorption and longer mixing time.

**Figure 19:**
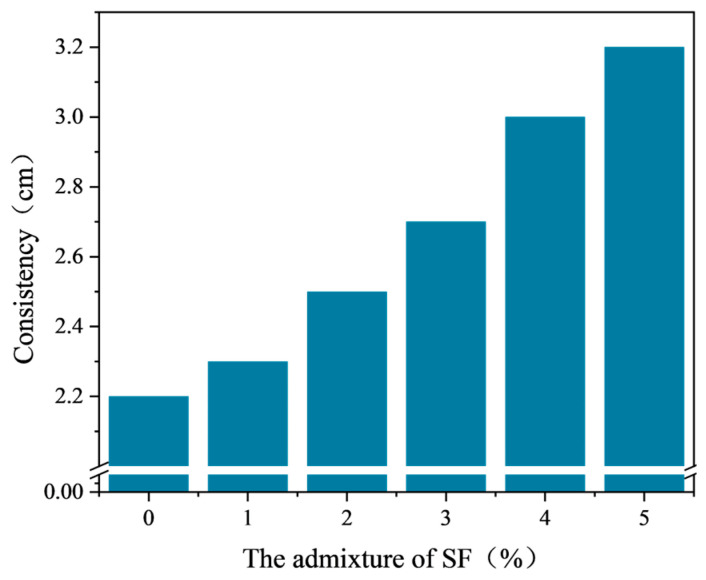
Consistency of emulsified asphalt mixture with different SF content.

After the viscosity experiment, the viscosity of emulsified asphalt mixtures with different SF contents was obtained as shown in Figure 19. According to the experimental data in Figure 19, when the SF content is within the range of 0% to 4%, the viscosity of the emulsified asphalt mixture is within the range of 2–3 cm, meeting the specification requirements. When the SF content is within the range of 0% to 5%, the viscosity gradually increases with the increase in SF content. When the SF content reaches 5%, the viscosity is 3.2 cm, an increase of 1 cm compared to the absence of SF, which does not meet the specification requirements.

In emulsified asphalt mixtures, the content of coarse particles increases with the gradual increase in SF content. As a result, the total specific surface area of the mineral material decreases, reducing the amount of water that can be adsorbed by the mineral material. Consequently, the amount of free water increases, improving the overall flowability of the emulsified asphalt mixture and increasing the viscosity. However, excessive free water makes the viscosity of the emulsified asphalt mixture too high, deteriorating the mixing performance and failing to meet the specification requirements.

From the results of the mixing and consistency tests, it can be concluded that although adding SF can improve the microwave absorption performance of the emulsified asphalt mixture, a larger dosage affects the mixing performance and greatly increases the economic cost. Therefore, the reasonable dosage range of SF in this study is 0% to 4%.

### 3.2. Microwave Absorption Performance Test of Emulsified Asphalt Mixture

#### 3.2.1. Evaluation Indicators for Microwave Absorption Performance

1. Temperature change ($\Delta_{T}$)

According to the theory of heat transfer, when there is a temperature difference between different objects or within the same object, energy transfer occurs through the collision of molecules, atoms, and electrons inside the object. When microwaves are used to heat an emulsified asphalt mixture, the temperature change is the most intuitive reflection of the mixture’s energy absorption. After absorbing microwaves, the temperature of the mixture increases, and the more microwave energy is absorbed, the greater the temperature change. Therefore, the temperature change in the mixture can be used to represent its microwave absorption performance. The calculation method for temperature change ($\Delta_{T}$) is shown in Equation (5).

(5) $$\Delta_{T}=T_{2}-T_{1}$$

where

$\Delta_{T}$ is temperature change, °C;

$T_{1}$ is the temperature of the mixture before microwave heating, °C;

$T_{2}$ is the temperature of the mixture after microwave heating, °C.

2. Temperature rise rate (VT)

When the mixture contains different microwave-absorbing materials, microwave heating will increase its temperature. In this case, the temperature change alone is not sufficient to fully reflect the magnitude of the microwave absorption performance. The rate of temperature rise is also needed to reflect the microwave absorption performance. When microwave-absorbing material is added to the emulsified asphalt mixture, a greater rate of temperature increase compared to when it is not added indicates that this material can improve the microwave absorption performance of the mixture. The calculation method for the temperature rise rate (VT) is shown in Equation (6).

(6) $$V_{T}=\frac{\Delta_{T}}{t}$$

where

$V_{T}$ is the temperature rise rate, °C/s;

*t* is the heating time, s.

The rate of temperature rise can be divided into two situations:

Linear Temperature Rise: When microwave heating causes the temperature of the mixture to change linearly with the heating time, the temperature rises linearly as the heating time increases. In this case, the slope of the temperature vs. time curve can reflect the rate of temperature increase.

Non-linear Temperature Rise: When microwave heating results in a non-linear relationship between the temperature of the mixture and the heating time, the temperature does not rise linearly as the heating time increases, and the slope of the curve changes continuously during the heating process. In this scenario, Equation (6) can be used to reflect the average temperature rise rate throughout the heating process.

3. The rate of increase in temperature rise rate (*ω*)

When an emulsified asphalt mixture contains microwave-absorbing materials of the same type but in different dosages, the temperature change caused by microwave heating may not be significantly different, and the rate of temperature increase may also be minimal. In this case, the rate of increase in the temperature rise rate can be used to reflect the change in microwave absorption performance. The calculation method for the increase rate of the temperature rise rate (*ω*) is shown in Equation (7).

(7) $$\omega =\frac{V_{T2}-V_{T1}}{V_{T1}}$$

where:

ω is the rate of increase in temperature rise rate, %;

$V_{T1}$ is the rate of temperature increase before the increase, °C/s;

$V_{T2}$ is the rate of temperature increase after enlargement, °C/s.

4. Quality change ($\Delta_{m}$)

When the emulsified asphalt mixture is heated by microwaves, it absorbs the microwave energy, causing its temperature to rise. As the temperature increases, the water in the system will gradually evaporate. When the internal temperature of the mixture exceeds 100 °C, a significant amount of water will evaporate. The demulsification process of emulsified asphalt involves the adhesion of asphalt particles to aggregates and the subsequent evaporation of water from the emulsified asphalt. Therefore, the change in mass of the emulsified asphalt mixture before and after heating can reflect the evaporation of water in the mixture. The stronger the microwave absorption ability of the emulsified asphalt mixture, the faster the evaporation rate of water in the mixture, and the greater the mass change in the mixture before and after heating, corresponding to a faster demulsification speed of the emulsified asphalt. The calculation method for the quality change quantity ($\Delta_{m}$) is shown in Equation (8).

(8) $$\Delta_{m}=m_{2}-m_{1}$$

where

$\Delta_{m}$ is the quality change quantity, g;

$m_{1}$ is the mass of the mixture before microwave heating, g;

$m_{2}$ is the mass of the mixture after microwave heating, g.

5. Quality reduction rate ($V_{m}$)

The rate of quality reduction can reflect the speed of mass reduction in the emulsified asphalt mixture during the heating process. The calculation method for the rate of mass reduction ($V_{m}$) is shown in Equation (9).

(9) $$V_{m}=\frac{\Delta_{m}}{t}$$

where $V_{m}$ is the quality reduction rate, g/s.

#### 3.2.2. Analysis of the Effect of SF Content on the Microwave Absorption Performance of SF-EAM

**Figure 20:**
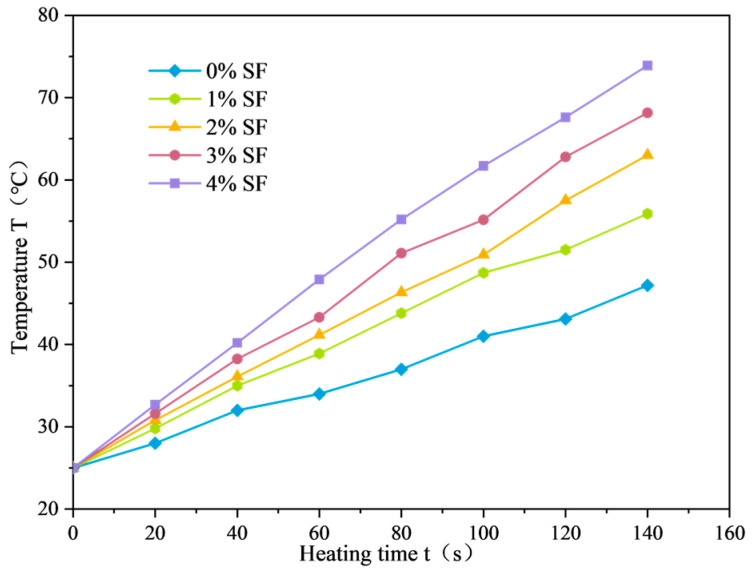
Temperature variation curve of SF-EAM (SF content).

**Figure 21:**
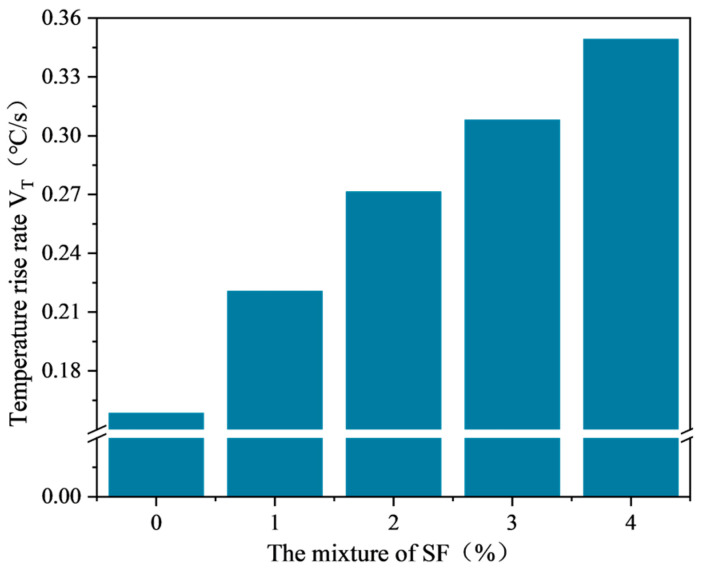
Relationship between temperature rise rate and SF content.

The average temperature variation curve of SF-EAM (silicon carbide-ferrite emulsified asphalt mixture) under microwave heating with different SF doping levels is shown in Figure 20. The relationship between the temperature rise rate and SF doping level is shown in Figure 21.

From Figure 20 and Figure 21, it can be seen that when the initial temperature is 25 °C, the microwave power is 200 W, and the SF ratio is 1:1, heating the emulsified asphalt mixture specimen with 0~4% SF for 140 s results in the surface temperature of the SF-EAM specimen continuously increasing with the duration of microwave radiation, showing a roughly linear relationship. As the SF content increases, the rate of temperature rise gradually increases. The increase in temperature rise rate is particularly significant when 1% SF is added. When 1%, 2%, 3%, and 4% SF were added, the increase rates of temperature rise rate were 39.0%, 70.4%, 93.7%, and 119.5%, respectively, compared to the absence of SF.

According to the thermal equilibrium conditions of the object, Equation (10) can be derived. Assuming that the microwave can be uniformly radiated to both the inside and outside of the specimen and that the electric field generated by the microwave is a uniform electric field, the electromagnetic parameters of the SF-EAM specimen remain unchanged, and the microwave absorption power of the specimen is constant. The maximum temperature of the specimen after 140 s of heating is 73.9 °C, with a temperature change that is not significant. During this process, the evaporation rate of water is slow, so the specimen can be regarded as an object with uniform and unchanged material properties throughout the entire heating process. Additionally, the thickness of the prepared specimen is relatively thin, allowing simultaneous heating of both the inside and outside, so internal heat transfer can be ignored. The divergence of heat flux density is zero, and the heat capacity of the specimen is constant. By substituting the above conditions into Equation (10) and solving the differential equation, Equation (11) can be obtained.

(10) $$divq=P-C\frac{\partial T}{\partial t}$$

where

*q* is the heat flux density, J/(m^2^·s)

*P* is the absorption power of an object to microwaves, W/m^3^;

*C* is the heat capacity, J/°C;

*T* is the temperature, °C;

*t* is the time, s.

(11) $$\frac{T}{t}=k$$

With the increase in SF content, the electromagnetic parameters of the emulsified asphalt mixture gradually increase, enhancing the microwave absorption performance of the mixture system. As a result, more microwaves can be absorbed in the same amount of time, causing the temperature rise rate to continue increasing. When SF is not added, the primary microwave-absorbing material in the mixture system is water. With the addition of 1% SF, both water and SF become the primary microwave-absorbing materials. The addition of SF significantly increases the electromagnetic parameters of the mixture, resulting in a substantial increase in the temperature rise rate.

**Figure 22:**
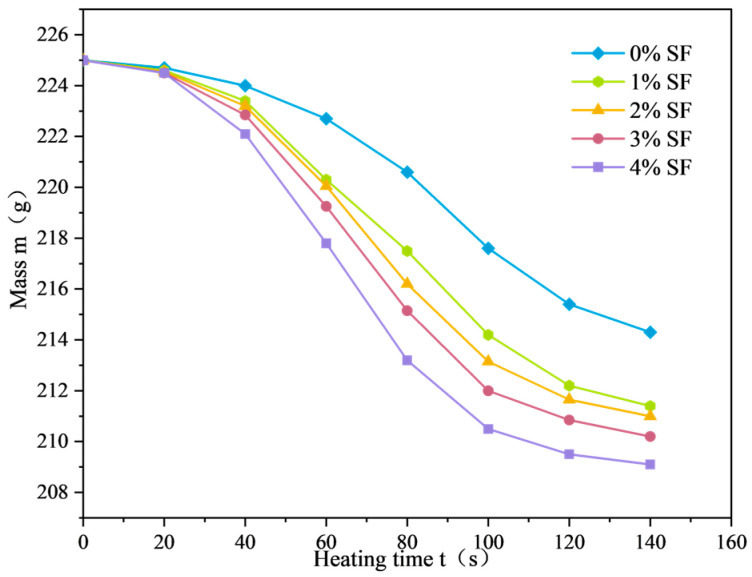
Quality change curve of SF-EAM (SF content).

The mass variation curves of SF-EAM under microwave heating with different SF dosages are shown in Figure 22. When the initial temperature is 25 °C, the microwave power is 1000 W, and the SF ratio is 1:1, and heating the emulsified asphalt mixture specimen with 0%~4% SF for 140 s causes the mass of the SF-EAM specimen to decrease continuously, showing a nonlinear relationship with the increase in microwave radiation time.

**Figure 23:**
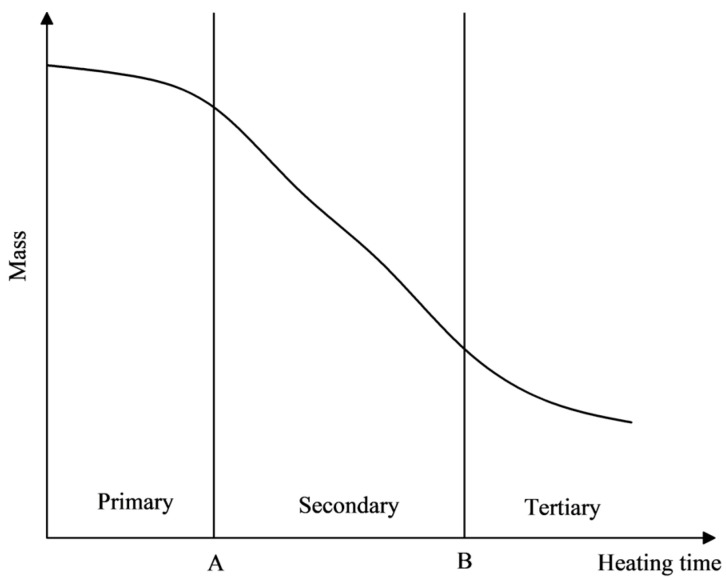
Curve of quality change law.

According to the pattern of the quality change curve, the process of quality reduction can be divided into three stages. The time separation node between the first and second stages is denoted as node A, and the time separation node between the second and third stages is denoted as node B, as shown in Figure 23.

The slope of the first stage curve is relatively small, and the rate of mass reduction ($V_{m}$) of the emulsified asphalt mixture specimen is low, with a small magnitude of mass reduction.

The slope of the second stage curve increases, and the rate of decrease in specimen mass ($V_{m}$) increases.

The third stage curve tends to flatten out, and the rate of decrease in specimen mass ($V_{m}$) decreases again, while the specimen mass remains stable and unchanged.

With the increase in SF content, the mass change ($\Delta_{m}$) of SF-EAM specimens continues to increase, the mass reduction rate ($V_{m}$) gradually increases, and nodes A and B continue to advance. When adding 1%, 2%, 3%, and 4% SF, the mass change increased by 27.1%, 30.8%, 38.3%, and 48.6%, respectively, compared to not adding SF.

The stages of the quality change process are described as follows:

Heat Accumulation Stage: During this stage, the microwave begins to irradiate the specimen. The internal temperature of the specimen rapidly increases, and the mixture remains at a relatively low temperature. The water evaporation rate is slow, and the rate of mass reduction ($V_{m}$) of the specimen is low.

Rapid Evaporation Stage: During this stage, the specimen continuously absorbs microwaves, and the internal temperature reaches the boiling point of water. A large amount of water evaporates outward from the mixture, increasing the rate of mass reduction ($V_{m}$).

Quality Stability Stage: During this stage, although the internal temperature of the specimen is high, the water in the mixture is eventually consumed, and the mass of the specimen stabilizes.

As the SF content increases, the temperature rise rate ($V_{T}$) of SF-EAM specimens increases, accelerating the water evaporation rate. Consequently, the mass reduction rate ($V_{m}$) continues to increase, with nodes A and B advancing continuously. The mass change in the specimen ($\Delta_{m}$) also increases with the increase in SF content. According to the results of temperature and quality testing, the optimal dosage of SF is 4%.

#### 3.2.3. Analysis of the Influence of SF Composite Ratio on the Microwave Absorption Performance of SF-EAM

**Figure 24:**
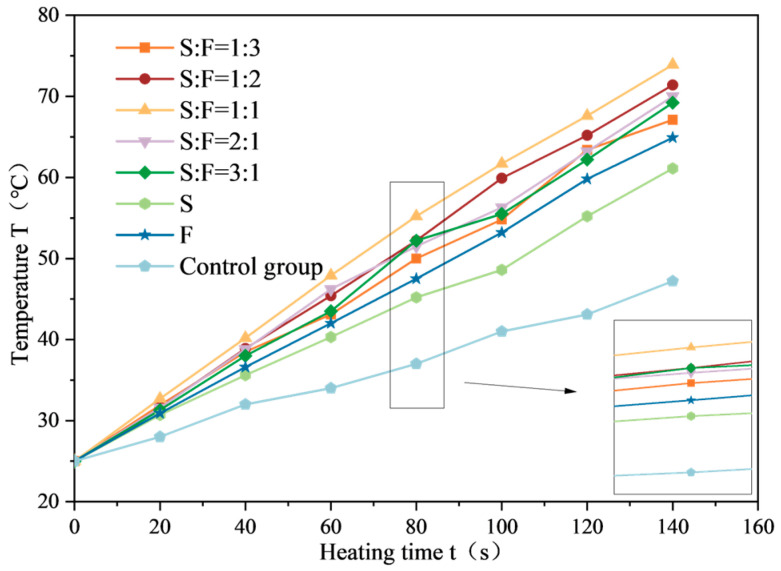
Temperature variation curve of SF-EAM (SF composite ratio).

**Figure 25:**
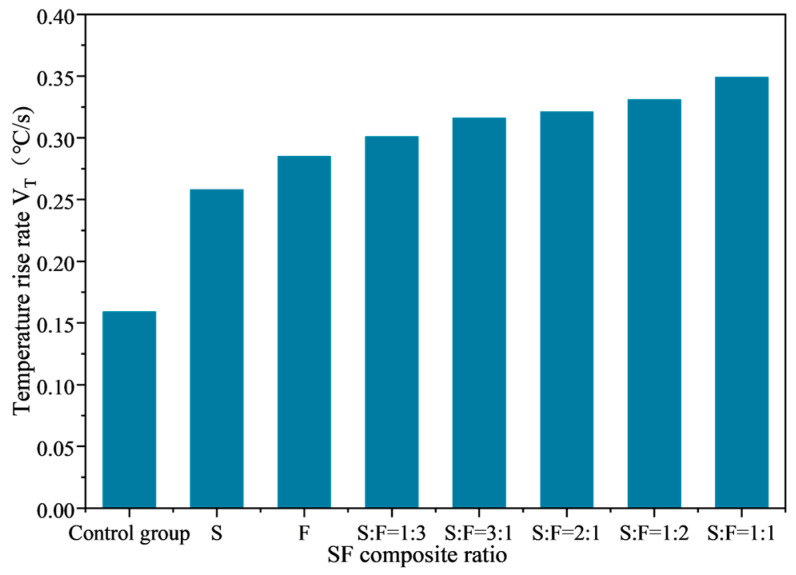
Relationship between temperature rise rate and SF composite ratio.

The average temperature variation curve of SF-EAM microwave-heated surfaces with different SF ratios is shown in Figure 24, and the relationship between temperature rise rate and SF composite ratio is shown in Figure 25.

From Figure 24 and Figure 25, it can be seen that when the initial temperature is 25 °C and the microwave power is 200 W, heating the emulsified asphalt mixture specimen with 4% SF for 140 s results in a significantly increased temperature rise rate ($V_{T}$) compared to the control group without microwave-absorbing material. The temperature rise rate ($V_{T}$) of the emulsified asphalt mixture specimen with either SiC or Fe_3_O_4_ single-phase material added is significantly increased, and the temperature rise rate ($V_{T}$) of the emulsified asphalt mixture specimen with SF added is greater than that of the mixture with single-phase material added ($V_{T}$).

When different proportions of SF are added, the temperature rise rates ($V_{T}$) from small to large are as follows:

S:F = 1:3, S:F = 3:1, S:F = 2:1, S:F = 1:2, S:F = 1:1.

Among these, the temperature rise rate ($V_{T}$) of emulsified asphalt mixture specimens with an SF ratio of S = 1:1 increased by 35.5% compared to the addition of SiC alone and increased by 22.6% compared to the addition of Fe_3_O_4_ alone.

These results indicate that a balanced composite ratio of SiC and Fe_3_O_4_ (1:1) provides the most effective microwave absorption performance, significantly enhancing the temperature rise rate of the emulsified asphalt mixture compared to single-phase materials.

Compared to doping SiC or Fe_3_O_4_ separately, doping SF can simultaneously leverage the electrical and magnetic loss mechanisms in the material, fully utilizing the electromagnetic energy in the microwave. Therefore, the microwave absorption effect of composite materials is better than that of single-phase materials. Under the above five composite ratios, the electromagnetic parameters of SF gradually increase, enhancing the microwave absorption performance of SF-EAM, and the temperature rise rate ($V_{T}$) will also gradually increase.

**Figure 26:**
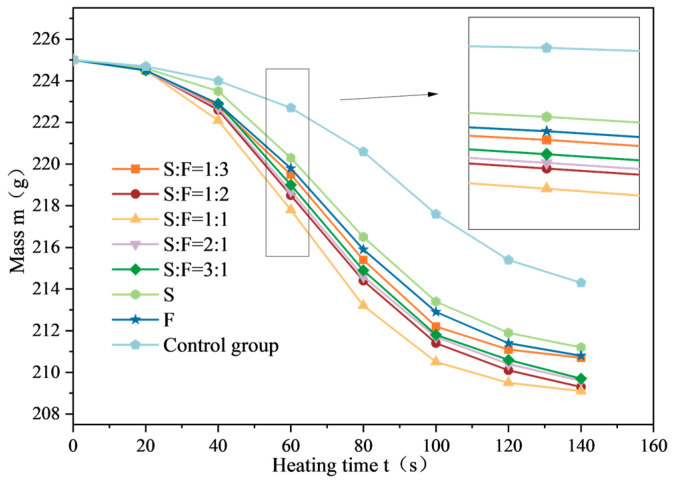
Quality change curve of SF-EAM (SF composite ratio).

The mass variation curves of SF-EAM under microwave heating with different SF ratios are shown in Figure 26. As illustrated, when the initial temperature is 25 °C and the microwave power is 1000 W, heating the emulsified asphalt mixture specimen with 4% SF for 140 s results in the highest temperature rise rate ($V_{T}$) at a composite ratio of S:F = 1:1. The mass change ($\Delta_{m}$) is also the largest, with nodes A and B showing the most significant advance. Although the timing of node A and node B in the quality change curve varies at different ratios, there is a trend towards consistency in the final quality of emulsified asphalt mixture specimens at three ratios: S:F = 2:1, S:F = 1:2, and S:F = 1:1. According to the results of temperature testing and quality testing, among the various composite ratios set in this study, the microwave absorption performance of SF-EAM is optimal when the composite ratio of SiC to Fe_3_O_4_ is 1:1.

#### 3.2.4. Analysis of the Influence of Environmental Temperature on the Microwave Absorption Performance of SF-EAM

**Figure 27:**
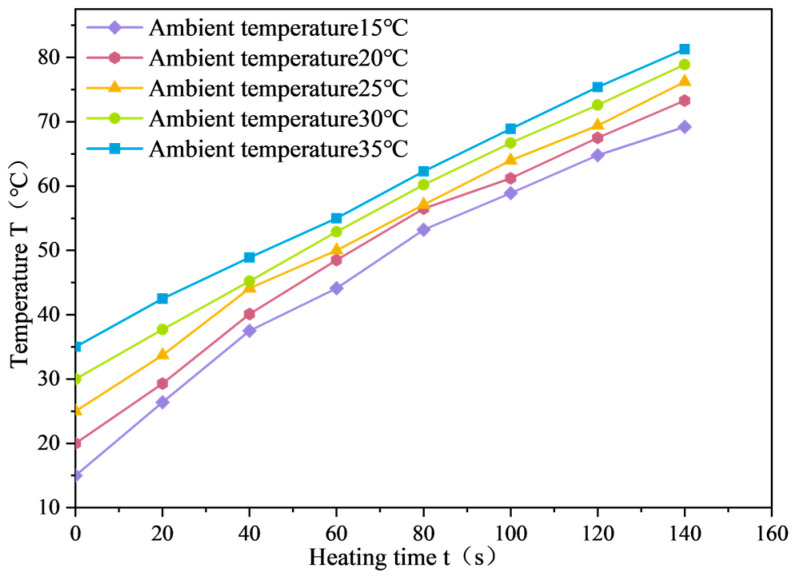
Temperature variation curves of SF-EAM under different ambient temperatures.

**Figure 28:**
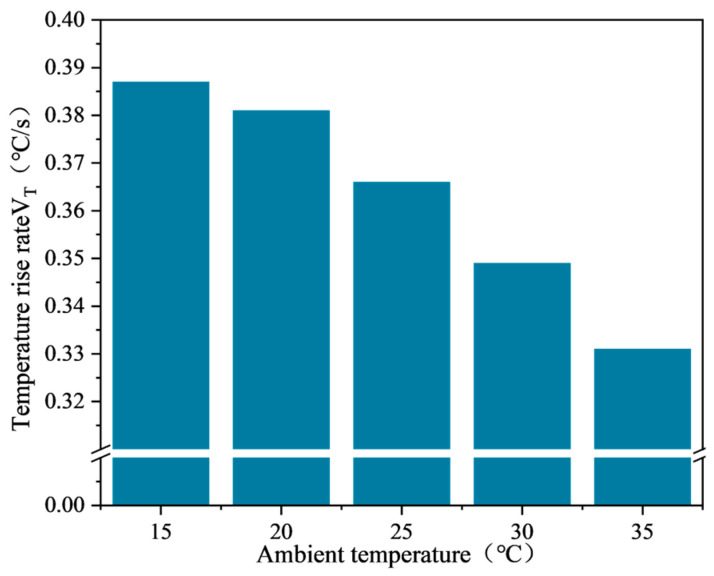
Relationship between temperature rise rate and ambient temperature.

The average surface temperature variation curve of SF-EAM heated by microwave at different ambient temperatures is shown in Figure 27, and the relationship between the temperature rise rate ($V_{T}$) and ambient temperature is shown in Figure 28.

According to Figure 27 and Figure 28, when the ambient temperature changes, heating the emulsified asphalt mixture specimen with 4% SF (S:F = 1:1) for 140 s results in a linear relationship between the average surface temperature (*T*) and heating time (*t*) of the emulsified asphalt mixture specimen at different ambient temperatures. As the ambient temperature continues to rise, the rate of temperature increase of emulsified asphalt mixture specimens decreases. When the ambient temperature is 15 °C, the rate of temperature increase ($V_{T}$) is 0.387 °C/s, and when the ambient temperature is 35 °C, the rate of temperature increase is 0.331 °C/s, a decrease of 14.5% compared to 15 °C.

These results indicate that higher ambient temperatures negatively impact the microwave absorption performance of the SF-EAM, leading to a lower rate of temperature increase.

The absorption power of an object to microwaves (P) is related to the microwave operating frequency (f) and microwave electric field strength (E), and is independent of the ambient temperature (T). Therefore, while ambient temperature does impact the microwave absorption performance of SF-EAM, the degree of influence is minimal. The rate of temperature increase ($V_{T}$) of SF-EAM varies at different temperatures due to the principles of heat transfer, where the larger the temperature difference between two media, the faster the transfer speed. When preparing the specimen, the lower the ambient temperature, the greater the temperature difference between the specimen and the air in the microwave field, leading to a faster heat transfer rate and a quicker temperature rise rate ($V_{T}$) of the specimen. Hence, the rate of temperature increase ($V_{T}$) at this time cannot be used as an indicator to evaluate the microwave absorption performance of SF-EAM.

**Figure 29:**
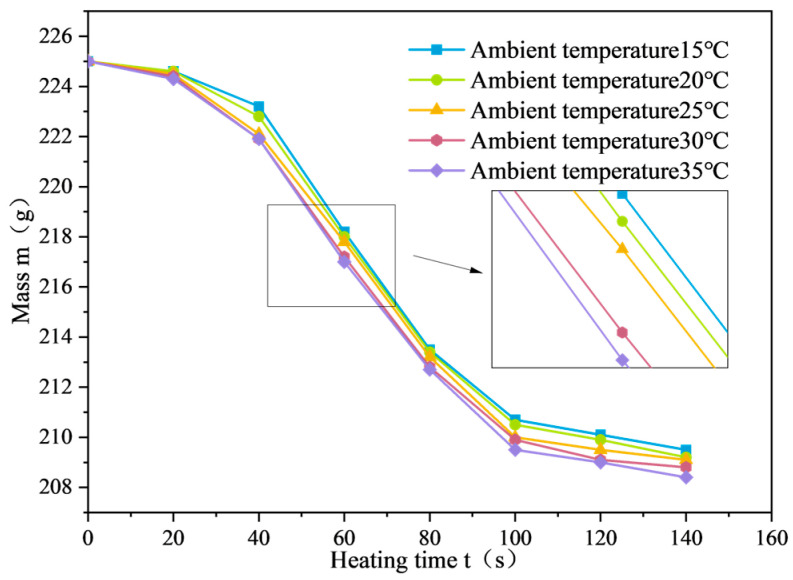
Mass change curve of SF-EAM at different ambient temperatures.

The mass change curve of SF-EAM under microwave heating at different ambient temperatures is shown in Figure 29. From Figure 29, it can be seen that when the ambient temperature changes, heating the emulsified asphalt mixture specimen with 4% SF (S:F = 1:1) for 140 s results in greater mass change ($\Delta_{m}$) at higher ambient temperatures, with node A appearing earlier in the mass change process. This indicates that the higher the ambient temperature, the better the microwave absorption performance of SF-EAM.

The conclusion drawn from the quality test results contrasts with that of the temperature test results because ambient temperature affects the water evaporation rate of the emulsified asphalt mixture and thus its quality change ($\Delta_{m}$). Therefore, at this time, the quality change ($\Delta_{m}$) cannot be used as an indicator to evaluate the microwave absorption performance of SF-EAM. According to the results of temperature and quality testing experiments, it can be concluded that while ambient temperature affects the temperature rise rate ($V_{T}$) and mass change ($\Delta_{m}$) of SF-EAM, it has little effect on the microwave absorption performance of SF-EAM.

Based on the data obtained from the experiments, the microwave absorption efficiency remains essentially unchanged with temperature variations, indicating that the microwave absorption efficiency is constant, and the impact of ambient temperature on microwave absorption performance is negligible. Although an increase in temperature can directly affect the degree of curing by influencing the evaporation of water, ambient temperature is a factor that is difficult to alter, and its economic significance in practical applications is not high. Therefore, this paper does not delve into the exploration of the impact of ambient temperature on microwave absorption performance.

#### 3.2.5. Analysis of the Influence of Microwave Power on the Microwave Absorption Performance of SF-EAM

**Figure 30:**
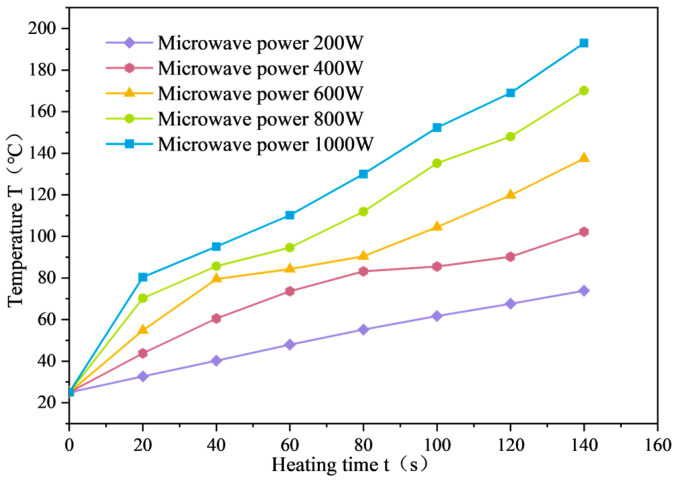
Temperature variation curve of SF-EAM under different powers.

**Figure 31:**
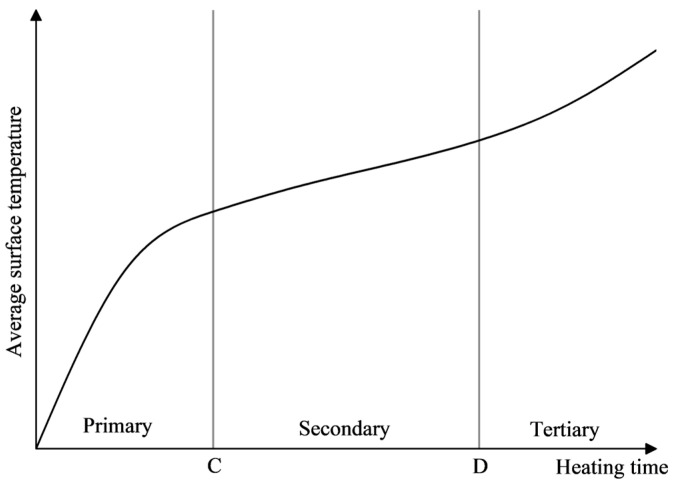
Surface average temperature variation curve.

The average surface temperature variation curve of SF-EAM under microwave heating at different powers is shown in Figure 30. It can be seen that at an initial temperature of 25 °C, the surface temperature (T) of the emulsified asphalt mixture specimen mixed with 4% SF (S:F= 1:1) varies linearly with the heating time (t) when heated for 140 s at 200 W. When the microwave power is 400–1000 W, the temperature (T) and heating time (t) exhibit a nonlinear relationship. The surface temperature growth trend of the specimen can be divided into three stages. The time separation node between the first and second stages is denoted as node C, and the time separation node between the second and third stages is denoted as node D, as shown in Figure 31.

In these stages:

The slope of the first-stage curve is the largest, and the rate of temperature rise ($V_{T}$) is the highest.

The second-stage curve is gentle, and the rate of temperature rise ($V_{T}$) is relatively small.

The slope of the third stage increases again but not as steeply as the first stage, and the rate of temperature rise ($V_{T}$) increases again.

As the microwave power increases, the rate of temperature rise ($V_{T}$) of emulsified asphalt mixture specimens continues to increase. When the microwave power is 200 W, 400 W, 600 W, 800 W, and 1000 W, the temperature change ($\Delta_{T}$) of the specimen heated for 140 s is 48.9 °C, 77.2 °C, 112.5 °C, 145.1 °C, and 168 °C, respectively.

**Figure 32:**
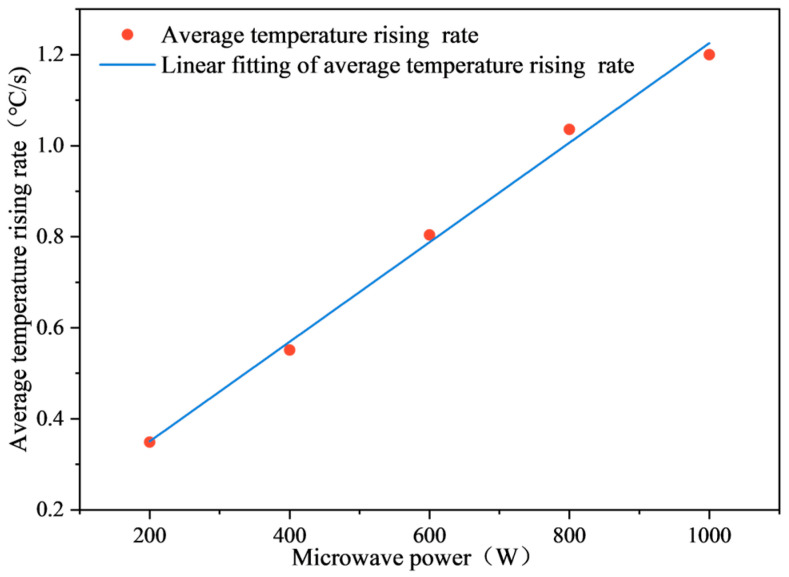
Relationship between average temperature rise rate and microwave power.

The relationship between the average temperature rise rate and microwave power is shown in Figure 32. According to Figure 32, the rate of average temperature rise is roughly proportional to microwave power. There are differences in the duration of the three stages of temperature changes in SF-EAM under different powers. As microwave power continues to increase, the rate of temperature rise ($V_{T}$) of emulsified asphalt mixture specimens in the three stages also increases. That is, the higher the microwave power, the shorter the duration of the first and second stages, and the earlier the C and D node times.

When the microwave power is 200 W, the temperature of the emulsified asphalt mixture specimen uniformly increases after heating for 140 s, indicating that the first 140 s are in the first stage. For microwave powers of 400 W, 600 W, 800 W, and 1000 W, the duration of the first stage is about 70 s, 50 s, 30 s, and 20 s, respectively, and the duration of the second stage is about 40 s, 35 s, 30 s, and 25 s, respectively.

The temperature variation in emulsified asphalt mixture specimens during microwave heating is closely related to the moisture content in the mixture. The process can be divided into three distinct stages:

Initial Heating Stage: During this stage, the water and SF (SiC-Fe_3_O_4_) in the emulsified asphalt mixture absorb microwaves together, resulting in a rapid increase in the specimen’s temperature.

Temperature Stabilization Stage: At this stage, the temperature rises above 100 °C, reaching the boiling point of water. A large amount of water evaporates in the mixture system, and the evaporation of water takes away most of the heat. This leads to a relatively slow rate of temperature increase ($V_{T}$).

Secondary Heating Stage: In this stage, the water in the emulsified asphalt mixture has almost completely evaporated. The heat carried away by the evaporation of water is not sufficient to prevent the temperature from rising. However, since only SF and a small amount of water absorb microwaves during this stage, the rate of temperature rise ($V_{T}$) is lower than in the initial heating stage.

The formula for electric power is shown in Equation (12), and the formula for electric field strength is shown in Equation (13).

(12) $$P_{a}=\frac{U^{2}}{R}$$

where

$P_{a}$ is the microwave power, W;

U is the potential difference between two points in an electric field, V;

R is the resistance, Ω.

(13) $$E=\frac{U}{d}$$

where

E is the electric field strength, V/m;

d is the distance between two points in an electric field, m.

(14) $$V_{T}\propto P_{a}$$

When the microwave power increases, the electric field strength (*E*) generated by electromagnetic waves also increases. According to Equations (12) and (13), assuming that the electric field generated by a microwave with a fixed frequency is a uniform electric field, and the distance between the resistance *R* and the electric field (*d*) remains constant, the square of the electric field strength (*E*) in the microwave is proportional to the microwave power *P*. Thus, it can be concluded that the absorption power ($P_{a}$) of the emulsified asphalt mixture specimen to microwaves is proportional to the microwave power (*P*). Based on the previous conclusions, it can also be concluded that the average temperature rise rate ($V_{T}$) of the specimen is roughly proportional to the microwave power (*P*), as shown in Equation (14).

**Figure 33:**
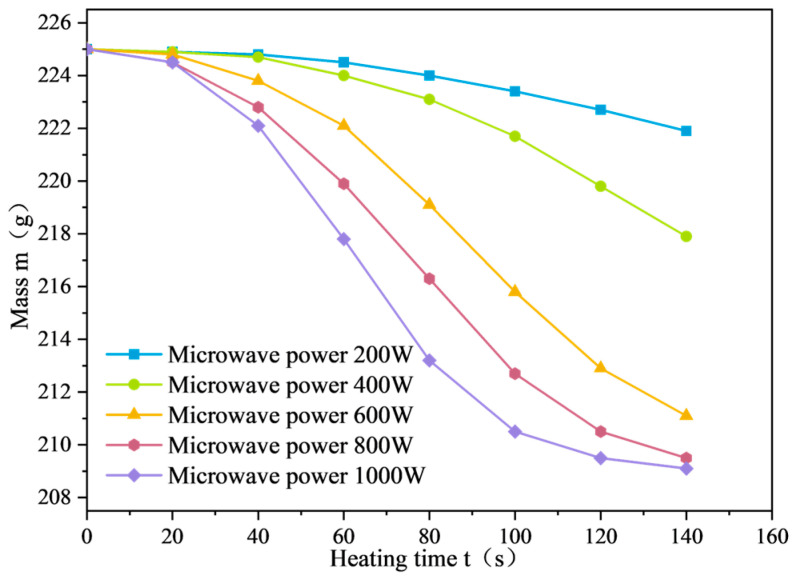
Mass change curve of SF-EAM at different powers.

The mass change curve of SF-EAM under microwave heating at different powers is shown in Figure 33. As illustrated, at an initial temperature of 25 °C, the emulsified asphalt mixture specimens with 4% SF (S:F= 1:1) were heated for 140 s at different powers. With the continuous increase in microwave power, the mass change ($\Delta_{m}$) of the emulsified asphalt mixture specimens also increased.

When the microwave power was 200 W, 400 W, 600 W, 800 W, and 1000 W, the mass change ($\Delta_{m}$) of the specimens heated for 140 s was 3.1 g, 7.1 g, 13.9 g, 15.5 g, and 15.9 g, respectively. The quality change trend of emulsified asphalt mixture specimens heated for 140 s varies slightly under different powers:

At 200 W, the mass of the emulsified asphalt mixture specimens slowly decreases.

At 400 W, the rate of mass reduction of the specimen slightly increases, but the trend remains consistent with that at 200 W power.

At powers exceeding 600 W, the rate of mass reduction of the specimen increases significantly and begins to show a trend of “three stages”.

When the microwave power is low, the internal temperature of the emulsified asphalt mixture specimen heated for 140 s does not reach 100 °C, resulting in a slow water evaporation rate and a small mass change ($\Delta_{m}$). When the microwave power is high, the quality change in the emulsified asphalt mixture specimens corresponds to the average surface temperature change.

The stages of the process are as follows:

Heat Accumulation Stage: The temperature rise rate ($V_{T}$) is high, but the mass decrease rate ($V_{m}$) is low.

Rapid Evaporation Stage: The temperature rise rate ($V_{T}$) decreases while the mass decrease rate ($V_{m}$) increases rapidly.

Mass Stabilization Stage: The temperature continues to rise, but the mass remains basically unchanged.

**Figure 34:**
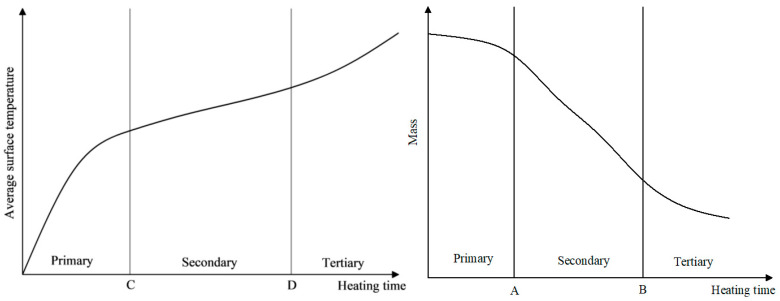
Corresponding relationship between temperature change and mass change.

Based on Figure 23 and Figure 31, the corresponding relationship between the temperature change process and the mass change process is shown in Figure 34. When heating SF-EAM with high power, the temperature and mass changes of SF-EAM are closely related to the moisture content in the material:

First Stage: The temperature rapidly increases while the mass remains almost unchanged.

Second Stage: The temperature exceeds the boiling point, and the mass rapidly decreases.

Third Stage: Water evaporation is completed, and the temperature continues to rise.

The second stage, where water evaporation occurs, is the microwave heating curing stage of SF-EAM. The microwave absorption performance of SF-EAM determines the development speed of the first two stages. The better the microwave absorption performance, the faster the development speed of these stages.

Comprehensive temperature and quality testing results indicate that when the microwave power exceeds 600 W, both the temperature and quality of SF-EAM undergo significant changes. Under the same conditions, higher microwave power results in better microwave absorption performance of SF-EAM. However, excessive microwave power can cause the temperature rise rate of SF-EAM to be too fast, making it difficult to control the microwave heating time and leading to resource waste.

Recommendation: This study recommends a microwave heating power range of 600–1000 W to optimize the microwave absorption performance of SF-EAM without causing excessive temperature rise rates and resource waste.

## 4. Conclusions

This article investigates the microwave absorption properties of SiC-Fe_3_O_4_ emulsified asphalt mixture (SF-EAM), and the main research conclusions are as follows:

(1) Gradation and Ratio Design: Based on the median value of MS-3 gradation, Gradation A was designed using the theory of the maximum density curve. Based on performance tests at the micro-surface, the ratio of each component of the emulsified asphalt mixture was determined as follows: mineral aggregate:modified emulsified asphalt:water:cement = 100:12.8:6:1.
(2) Effect of SF Addition on Mixing Performance: The addition of SF affects the mixing performance of the emulsified asphalt mixture. As the SF content increases, the mixing time and viscosity of the emulsified asphalt mixture also increase. The optimal dosage range of SF, obtained through mixing performance tests, is 0% to 4%.
(3) Microwave Absorption Performance: When the SF content is in the range of 0% to 4%, higher content results in better microwave absorption performance of SF-EAM. The optimal dosage of SF is 4%. When the composite ratio of SF is S:F = 1:1, the microwave absorption performance of SF-EAM is best.
(4) Influence of Environmental Temperature and Microwave Power: The environmental temperature has a minimal effect on the microwave absorption performance of the emulsified asphalt mixture. In contrast, microwave power has a significant impact. The higher the microwave power, the better the microwave absorption performance of SF-EAM. Considering both heating efficiency and economic factors, the recommended range of microwave power in this study is 600–1000 W.

## Figures and Tables

**Table 1:** Characteristics of various microwave-absorbing materials.

Specimen	Characteristics of Absorbing Materials	Deficiency	Reference
Steel fiber	Electric Loss Type, Magnetic Loss Type	High costs and technical requirements	Yang et al. [15]
Sulfurous iron ore slag	Magnetic Loss Type	High technical requirements	Zhang et al. [16]
Carbon fiber	Electric Loss Type	High costs	Wang et al. [17]
Steel slag	Magnetic Loss Type	Complex composition of steel slag	Wang [18]
Natural magnetite	Magnetic Loss Type	Thermal efficiency is related to the grade of Fe_3_O_4_	Guan et al. [19]
Fe_3_O_4_	Magnetic Loss Type	Easy to reunite, high technical requirements	Guo et al. [20]
Boron-doped SiC	Electric Loss Type	High technical requirements	Chen et al. [7]
SiC-Fe_3_O_4_	Electric Loss Type, Magnetic Loss Type	High costs	Liu Xiaoming [21]

**Table 2:** Technical performance of modified emulsified asphalt.

Test Item		Unit	Standard Specifications [22]	Test Result	Test Method
Remainder on sieve (1.18 mm sieve)		%	≤0.1	0.05	T0652
Electric charge		/	Positively charged cation	Positively charged cation	T0653
Engler viscosity E_25_		/	3~30	18	T0622
Asphalt standard viscosity C_25_		s	12~60	35	T0621
Evaporation residue content		%	≥60	65	T0651
Nature of evaporation residues	Penetration degree	0.1 mm	40~100	80	T0604
	Softening point	°C	≥53	66	T0606
	Ductility (5 °C)	cm	≥20	30	T0605
	Solubility	%	≥97.5	98	T0607
Storage stability	1 d	%	≤1	0.5	T0655
	5 d	%	≤5	2.5	

**Table 3:** Technical performance of mineral materials.

Specimen	Test Item	Unit	Standard Specifications [16]	Test Result	Test Method
Coarse aggregate	Crushing value	%	≤26	15	T0316
	LLA	%	≤28	20	T0317
	Polished stone value	BPN	≥42	50	T0321
	Sturdiness	%	≤12	7	T0314
	Flat and elongated particles	%	≤15	10	T0312
Fine aggregate	Sturdiness	%	≤12	6	T0340
Mineral	Sand equivalent	%	≥65	78	T0334

**Table 4:** Technical performance of mineral powder.

Test Item	Unit	Standard Specifications [23]	Test Result	Test Method
Apparent relative density	t/m^3^	≥2.50	2.8	T0352
Water content	%	≤1	0.8	T0103
Particle size range < 0.6 mm <0.15 mm <0.075 mm	% % %	100 90~100 75~100	100 98 92	T0351
Hydrophilicity	/	<1	0.8	T0353
Plasticity index	/	<4	3	T0354

**Table 5:** Technical properties of cement.

Test Item		Unit	Standard Specifications [24]	Test Result	Test Method
Setting time	Initial setting	min	≥45	132	T0505
	Final setting	min	≤600	270	
Compressive strength	3 d	MPa	≥17.0	20.4	T0553
	28 d	MPa	≥42.5	47.6	
Rupture strength	3 d	MPa	≥4.0	5.3	T0558
	28 d	MPa	≥7.0	8.2	
Thermal stability		/	Measurement records	Pass	T0505

**Table 6:** Technical performance of SiC.

Test Item	Unit	Standard Specifications [23]	Test Result	Test Method
Apparent relative density	t/m^3^	≥2.50	3.1	T0352
Water content	%	≤1	0.01	T0103
Particle size range < 0.6 mm <0.15 mm <0.075 mm	% % %	100 90~100 75~100	100 100 95	T0351
Hydrophilicity	/	<1	0.4	T0353
Plasticity index	/	<4	3	T0354

**Table 7:** Technical performance of Fe_3_O_4_.

Test Item	Unit	Standard Specifications [23]	Test Result	Test Method
Apparent relative density	t/m^3^	≥2.50	4.7	T0352
Water content	%	≤1	0.05	T0103
Particle size range < 0.6 mm <0.15 mm <0.075 mm	% % %	100 90~100 75~100	100 100 95	T0351
Hydrophilicity	/	<1	0.6	T0353
Plasticity index	/	<4	2.5	T0354

**Table 8:** Material usage ratio for cohesion test.

Specimen	Minerals	Water	Cement	Modified Emulsified Asphalt
Percentage of mineral mass	100%	1%	6%	10%, 11%, 12%, 13%, 14%

**Table 9:** Cumulative passing rate of each particle size.

Particle Size (mm)	9.5	4.75	2.36	1.18	0.6	0.3	0.15	0.075
Tebow equation *n* = 0.4	100	75.79	57.29	43.42	33.13	25.10	19.03	14.42
Median value of MS-3 grade	100	80	57.5	39	26.5	18.5	12.5	10

**Table 10:** Comparison of median data between calculated grading and MS-3 grading.

Particle Size Range (mm)	Passage Rate between Sieve Holes (%)		Difference (%)	Percentage of Difference (%)
	Tebow Equation *n* = 0.4	Median Value of MS-3 Grade		
4.75~9.5	24.21	20	−4.21	4.75~9.5
2.36~4.75	18.50	22.5	4.00	2.36~4.75
1.18~2.36	13.87	18.5	4.63	1.18~2.36
0.6~1.18	10.29	12.5	2.21	0.6~1.18
0.3~0.6	8.03	8	−0.03	0.3~0.6
0.15~0.3	6.07	6	−0.07	0.15~0.3
0.075~0.15	4.61	2.5	−2.11	0.075~0.15
Under 0.075	14.42	10	−4.42	Under 0.075

**Table 11:** Range of common materials used in emulsified asphalt mixtures.

Test Item	Unit	MS-3
Thickness after regimen	mm	8~10
Mineral dosage	kg/m^2^	10.0~22.0
Oil–stone ratio	%	6.0~8.5
Amount of cement, slaked lime (percentage of mineral mass)	%	0~3
Amount of water added (percentage of dry mineral mass)	%	Determined by the consistency of the mix

**Table 12:** Design indicators for mixing test and cohesion test.

Test Item		Standard Specifications [22]	Test Method
Mixable time (25 °C)		≥120 s	T0757
Adhesion test	30 min (Initial setting)	≥1.2 N·m	Adhesion test
	60 min (Open to traffic)	≥2.0 N·m	

**Table 13:** Preliminary proportions of emulsified asphalt mixtures.

Specimen	Minerals	Water	Cement	Modified Emulsified Asphalt
Percentage of mineral mass	100%	6%	1%	11%, 12%, 13%, 14%

**Table 14:** Design indicators for wet wheel wear test and load wheel sand adhesion test.

Item		Standard Specifications [22]	Test Method
Sand adhesion		≤450 g/m^2^	T0755
Wet track abrasion value	Immersion in water for 1 h	≤540 g/m^2^	Wet track abrasion value
	Immersion in water for 6 d	≤800 g/m^2^	

**Table 15:** Design indicators for mixing test and consistency test.

Test Item	Standard Specifications [22]	Test Method
Mixable time	≥120 s	T0757
Consistency	2~3 cm	T0751

## Data Availability

The original contributions presented in the study are included in the article, further inquiries can be directed to the corresponding author.
